# Revision and description of six species of *Choeradoplana* (Platyhelminthes, Tricladida), with an emendation to the genus

**DOI:** 10.3897/zookeys.1016.59617

**Published:** 2021-02-11

**Authors:** Domingo Lago-Barcia, Marcos Santos Silva, Fernando Carbayo

**Affiliations:** 1 Laboratório de Ecologia e Evolução, Escola de Artes, Ciências e Humanidades (EACH), Universidade de São Paulo (USP), Av. Arlindo Bettio, 1000, São Paulo, SP, 03828-000, Brazil; 2 Departamento de Zoologia, Instituto de Biociências, Universidade de São Paulo (USP), Rua do Matão, Tv. 14, 321, São Paulo, SP, 05508-090, Brazil; 3 Programa de Pós-Graduação em Sistemática, Taxonomia Animal e Biodiversidade, Museu de Zoologia, Universidade de São Paulo, São Paulo, SP, Brazil

**Keywords:** COI, flatworms, Geoplaninae, land planarians, rolled up, taxonomy

## Abstract

Living representatives of the Neotropical genus *Choeradoplana* Graff, 1896 (Geoplaninae, Tricladida, Platyhelminthes) are easily recognized by the typical shape of the head which is laterally expanded, rolled-up, and ventrally provided with two glandular cushions. In this study, the morphology and phylogeny (cytochrome C oxidase subunit I gene) of several species of land planarians are taxonomically investigated. Four of the six species studied are new to science, namely: *Ch. eudoxiae* Silva & Carbayo, **sp. nov.**, *Ch. claudioi* Lago-Barcia & Carbayo, **sp. nov.**, *Ch. onae* Lago-Barcia & Carbayo, **sp. nov.**, and *Ch. riutortae* Lago-Barcia & Carbayo, **sp. nov.** The species *Choeradoplana albonigra* and *Ch. eudoxiae* deviate from the usual body shape pattern in that the head does not present lateral expansions nor glandular cushions, becoming indistinguishable from its sister genus *Cephaloflexa*. *Pseudogeoplana tristriata* (Schultze & Müller, 1857) is also redescribed from a newly collected specimen and was discovered to be a member of *Choeradoplana*. Graff (1899) also studied another specimen that was considered to be conspecific with *P. tristriata*; however, in this new it is concluded that it is not conspecific but rather a new species. The name *Pseudogeoplana aevipandemiae* Lago-Barcia & Carbayo, **sp. nov.** is suggested for Graff’s specimen.

## Introduction

Members of the Neotropical land planarians genera *Choeradoplana* and *Cephaloflexa* (Platyhelminthes: Geoplanidae: Geoplaninae) can be easily ascribed to either genus based on the head shape, which is characteristically kept rolled up backwards in both genera. The two genera can be distinguished from each other in that the cephalic region in *Choeradoplana* is laterally expanded and ventrally provided with two glandular cushions separated by a longitudinal groove, whereas the cephalic region in *Cephaloflexa* is ventrally concave and the anterior third of the body becomes thinner very gradually.

In a recent multi-gene phylogenetic analysis of the Geoplaninae, representatives of a species preliminarily ascribed in the field to *Cephaloflexa* turned out to be nested in the *Choeradoplana* clade (Carbayo et al. 2013); therefore, the authors transferred it to that genus and the species is currently named *Choeradoplana albonigra* (Riester, 1938). However, the authors did not provide any morphological evidence.

In this paper, we describe or redescribe six species of Brazilian land planarians of the genus *Choeradoplana*. The external aspect of two of these species is similar to that of *Cephaloflexa*, namely the above-mentioned *C. albonigra*, and a new species described herein. We also studied a greenish individual recently collected and identified it as *Pseudogeoplana tristriata* (Schultze & Müller, 1857). The remaining four species were also collected recently and are new to science. We sequenced a fragment of the cytochrome C oxidase subunit I gene (COI) of each specimen to infer the phylogenetic relationships of the species treated herein, and the other representatives of the genus available in the GenBank.

## Materials and methods

### Molecular analysis

Each specimen was divided into two tissue portions, one for histology and one DNA extraction, respectively (see also section below ‘Morphological analysis’). Animal tissue destined for molecular studies was fixed in absolute ethanol and preserved at -20 °C. Genomic DNA was extracted using a standard ammonium acetate extraction protocol modified from Miller et al. (1988). The mitochondrial cytochrome oxidase I gene (COI) was amplified using the primers BarS (Álvarez-Presas et al. 2011), FlatwormCOIF (Sunnucks et al. 2006), and FlatwormCOIR (Lázaro et al. 2009). Standard PCR reactions were performed using Go Taq DNA polymerase (Promega) in a total volume of 25 µl, which included 0.5 ml of each primer (10 mM) and 2 ml of template DNA. PCR conditions for the COI gene consisted of an initial denaturation at 98 °C for 3 min, followed by 35 cycles of denaturation at 98 °C for 10 sec, annealing at 45 °C for 30 sec, end extension at 72 °C for 1 min, and a final extension at 72 °C for 5 min. Amplification products (~ 750 bp) were purified using ExoSAP-IT (Affymetrix, OH) before sequencing both strands on a 3730 DNA Analyzer (Thermo Fisher Scientific). DNA sequence data were edited using BioEdit 5.0.9 (Hall 1999) and aligned using the online version of the Mafft v7 software program (Katoh et al. 2019) using the G-INS-i algorithm. The sequences of the mitochondrial COI gene reported in this paper were deposited in the NCBI/GenBank data libraries under accession numbers MW127833-MW127842 and MW148795.

The phylogenetic analysis included the newly obtained partial nucleotide sequences for the COI gene (Table 1), and additional sequences of the 12 species of *Choeradoplana* available in the NCBI/GenBank data libraries (Table 1) (Álvarez-Presas et al. 2011; Carbayo et al. 2013; Álvarez-Presas et al. 2014; Lemos et al. 2014; Carbayo et al. 2018). Two sequences belonging to the genus *Matuxia* (Carbayo et al. 2013) were selected as the outgroup (Table 1). PartitionFinder2 ver. 2.1.1 software (Lanfear et al. 2016) on XSEDE (CIPRES Gateway to Science (Miller et al. 2010) was used to discover the partitions and the evolutionary model which best fit the nucleotide dataset under the Akaike Information Criterion corrected (AICc) (Hurvich and Tsai 1989). We estimated the phylogenetic relationships with a Bayesian Inference (BI) obtained using MrBayes v. 3.2.7 (Ronquist et al. 2012). The Markov chain Monte Carlo search was run up to 10,000,000 generations and stoprule activated to stopval = 0.01. Samples were taken every 10,000 generations, discarding the first 25% trees as burn-in, after which the chain reached stationarity, which ensured that the average split frequencies between the runs were less than 1%. Maximum Likelihood (ML) was implemented in RaxML 7.2.+ software (Stamatakis 2006) available on the CIPRES Science Gateway platform (Miller et al. 2010). Bootstrap support values (Felsenstein 1985) were obtained from 10,000 replicates in ML analyses. The final trees were viewed and edited on the FigTree v. 1.4.2 software program (http://tree.bio.ed.ac.uk/software/figtree/).

### Morphological analysis

All specimens included in this study (except for the holotype SMF No. 702, *Ch. albonigra*) (Table 1) were collected between May 2008 and January 2010 from conservation areas located in the southern region of the Atlantic Forest. Specimens were manually collected during daylight hours from under rocks, fallen leaves, and logs, and during nighttime hours when the animals are more active. Slides of the holotype of *Ch. albonigra* were obtained on loan from the Museum der Senckenbergischen Naturforschenden Gesellschaft in Frankfurt (**SMF**).

The flatworms were examined and photographed in vivo before being killed with boiling water. Tissue portions destined for histology were fixed in 10% formaldehyde and preserved in 80% ethanol. Color descriptions of the body of living and preserved specimens follow the online palette RAL colors (RAL gemeinnützige GmbH, available at https://www.ral-farben.de/uebersicht-ral-classic-farben.html?&L=1). Body portions were progressively dehydrated in an ascending series of ethanol and cleared in clove oil. Eye distribution was assessed from specimens cleared in clove oil. Body portions were subsequently embedded in Paraplast Tissue Embedding Medium and the resulting blocks were sectioned at 5–7 μm intervals using a retracting rotary microtome. Ribbons of embedding medium were affixed with albumin-glycerol (1:1) on glass slides placed on a hot plate and stained with the method Mallory-Heidenhain as modified by Cason (1950). Reconstructions of the copulatory apparatus were carried out from the histological sections using a camera lucida attached to a microscope. The relative thickness of cutaneous musculature was measured as a value relative to the body height in sections of the pre-pharyngeal region; space between normal and sunken longitudinal cutaneous layers was excluded from measurement. Specimens are deposited in the Museu de Zoologia da Universidade de São Paulo (**MZUSP**).

**Table 1.** List of samples used in this study showing species name, field number, museum accession number, type specimen status, collection locality and date, and GenBank accession number.

Species	Field number	Accession number	Type specimen	Coll. locality in Brazil	Coll. date	GenBank acc. (COI)
*Choeradoplana abaiba* Carbayo et al., 2018	F3905	MZUSP PL 1170	–	Parque Estadual da Serra do Tabuleiro, SC	07/14/09	MF802634
	F3894	MZUSP PL 1169	–	Parque Estadual da Serra do Tabuleiro, SC	07/13/09	MF802633
	F3865	MZUSP PL 1167	–	Parque Estadual da Serra do Tabuleiro, SC	07/12/09	MF802631
	F3866	MZUSP PL 1168	–	Parque Estadual da Serra do Tabuleiro, SC	07/12/09	MF802632
	F3864	MZUSP PL 1166	Holotype	Parque Estadual da Serra do Tabuleiro, SC	07/12/09	MF802630
	F3312	MZUSP PL 509	Paratype	Parque Estadual da Serra do Tabuleiro, SC	01/17/09	MF802629
	F3270	MZUSP PL 505	–	Parque Estadual da Serra do Tabuleiro, SC	01/15/09	MF802628
	F3315	MZUSP PL 511	–	Parque Estadual da Serra do Tabuleiro, SC	01/17/09	HQ542891
*Choeradoplana agua* Carbayo et al., 2018	F2214	MZUSP PL 448	–	Parque Estadual do Desengano, RJ	03/19/08	MF802637
	F2205	MZUSP PL 446	Paratype	Parque Estadual do Desengano, RJ	03/18/08	MF802636
	F2204	MZUSP PL 445	Paratype	Parque Estadual do Desengano, RJ	03/18/08	MF802635
	F2205	MZUSP PL 446	Paratype	Parque Estadual do Desengano, RJ	03/18/08	KF971686
	F3952	MZUSP PL 619	Paratype	Parque Estadual do Desengano, RJ	08/09/08	KF971680
*Choeradoplana albonigra* (Riester, 1938)	SMF N°702	–	Holotype	Teresópolis, RJ	04/09/14	–
	F2313	MZUSP PL 1109	–	Reserva Biológica Augusto Ruschi, ES	26/05/08	KF971684
	F2391	MZUSP PL 1113	–	Reserva Biológica Augusto Ruschi, ES	27/05/08	KF971683
	F3991	MZUSP PL 2273	–	Parque Estadual do Desengano, RJ	08/10/09	–
	F4024	MZUSP PL 2152	–	Parque Estadual do Desengano, RJ	08/11/09	–
	F4031	MZUSP PL 2153	–	Parque Estadual do Desengano, RJ	08/11/09	–
	F4081	MZUSP PL 1083	–	Parque Estadual do Desengano, RJ	08/13/09	KC608327
*Choeradoplana banga* Carbayo & Froehlich, 2012	F3706	MZUSP PL 568	–	Parque Estadual da Cantareira, SP	04/19/09	MF802639
	F3006	MZUSP PL 1001	Paratype	Parque Estadual da Cantareira, SP	12/14/08	MF802638
	F2023	MZUSP PL 1000	Holotype	Parque Estadual da Cantareira, SP	01/30/08	KC608267
	F3011	MZUSP PL 1002	Paratype	Parque Estadual da Cantareira, SP	12/14/08	KC608301
*Choeradoplana benyai* Lemos & Leal-Zanchet, 2014	F3813	MZUSP PL 1165	–	Parque Estadual da Serra do Tabuleiro, SC	07/12/09	MF802641
	F3494	MZUSP PL 1163	–	Floresta Nacional de São Francisco de Paula, RS	01/28/09	MF802640
	-	MZU PL.00151	Paratype	Floresta Nacional de São Francisco de Paula, RS	03/21/10	KJ690049
*Choeradoplana* sp.	F2332	MZUSP PL 2276	–	Reserva Biológica Augusto Ruschi, ES	26/05/08	MW127841*
	F2390	MZUSP PL 1155	–	Reserva Biológica Augusto Ruschi, ES	27/05/08	MW127842*
*Choeradoplana bocaina* Carbayo & Froehlich, 2012	F2822	MZUSP PL 997	Holotype	Parque Nacional da Serra da Bocaina, SP	09/08/09	KC608288
	F2104	MZUSP PL 999	Paratype	Parque Nacional da Serra da Bocaina, SP	02/10/08	KC608273
	F2803	MZUSP PL 998	Paratype	Parque Nacional da Serra da Bocaina, SP	09/07/08	KC608283
*Choeradoplana claudioi* sp. nov.	F2424	MZUSP PL 1156	Holotype	Reserva Biológica Augusto Ruschi, ES	28/05/08	MW127839*
	F2510	MZUSP PL 1157	Paratype	Reserva Biológica Augusto Ruschi, ES	29/05/08	MW127840*
*Choeradoplana eudoxiae* Silva & Carbayo, sp. nov.	F3417	MZUSP PL 2272	Holotype	Floresta Nacional de São Francisco de Paula, RS	01/22/09	–
*Choeradoplana gladismarie* Carbayo et al. 2013	F3802	MZUSP PL 1004	Paratype	Parque Estadual Intervales, SP	07/07/09	KC608326
	F3092	MZUSP PL 1003	Holotype	Parque Estadual Intervales, SP	12/12/08	KC608306
*Choeradoplana iheringi* Graff, 1899	F3465	MZUSP PL 537	–	Floresta Nacional de São Francisco de Paula, RS	01/25/09	MF802660
	F3454	MZUSP PL 535	–	Floresta Nacional de São Francisco de Paula, RS	01/25/09	MF802658
	F3447	MZUSP PL 531	–	Floresta Nacional de São Francisco de Paula, RS	01/25/09	MF802654
*Choeradoplana iheringi* Graff, 1899	F3430	MZUSP PL 528	–	Floresta Nacional de São Francisco de Paula, RS	01/23/09	MF802651
	F3409	MZUSP PL 524	–	Floresta Nacional de São Francisco de Paula, RS	01/22/09	MF802649
	-	MZU PL.00156	–	?		KJ690046
*Choeradoplana marthae* Froehlich, 1955	F2137	MZUSP PL 1153	–	Estação Biológica de Boraceia, SP	02/14/08	MF802665
*Choeradoplana minima* Lemos & Leal-Zanchet, 2014	-	MZU PL.00143	Paratype	Floresta Nacional de São Francisco de Paula, RS	02/25/10	KJ690052
	-	MZU PL.00145	Paratype	Floresta Nacional de São Francisco de Paula, RS	06/09/11	KJ690051
*Choeradoplana onae* Lago-Barcia & Carbayo, sp. nov.	F2230	MZUSP PL 2267	Paratype	Reserva Biologica de Ruschi, SP	05/24/08	MW127834*
	F2235	MZUSP PL 2277	–	Reserva Biologica de Ruschi, SP	05/24/08	MW127837*
	F2281	MZUSP PL 2268	Paratype	Reserva Biologica de Ruschi, SP	26/05/08	MW127836*
	F2310	MZUSP PL 2269	Paratype	Reserva Biologica de Ruschi, SP	26/05/08	MW127838*
	F2414	MZUSP PL 2270	Holotype	Reserva Biologica de Ruschi, SP	27/05/08	MW127835*
	F2311	MZUSP PL 1108	–	Reserva Biologica de Ruschi, SP	26/05/08	KF971685
*Choeradoplana pucupucu* Carbayo et al., 2018	F2844	MZUSP PL 541	Holotype	Parque Nacional da Serra da Bocaina, SP	09/01/08	MF802666
	F2840	MZUSP PL 540	Paratype	Parque Nacional da Serra da Bocaina, SP	09/20/08	KC608293
*Choeradoplana riutortae* Lago-Barcia & Carbayo, sp. nov.	F4217	MZUSP PL 1174	Paratype	Parque Nacional da Serra dos Orgãos, RJ	01/06/10	MW148795 *
	F4218	MZUSP PL 2274	Holotype	Parque Nacional da Serra dos Orgãos, RJ	01/06/10	–
	F4261	MZUSP PL 2275	Paratype	Parque Nacional da Serra dos Orgãos, RJ	01/08/10	–
*Choeradoplana tristriata* (Schultze & Müller, 1857)	F3226	MZUSP PL 2271	–	Parque Estadual da Serra do Tabuleiro, SC	01/11/09	MW127833*
*Matuxia matuta* (Froehlich, 1955)	F2184	MZUSP PL 1021	–	Parque Estadual do Desengano, RJ	03/17/08	KC608276
	F2187	MZUSP PL 1022	–	Parque Estadual do Desengano, RJ	03/17/08	KC608277
*Choeradoplana* sp. (in Álvarez-Presas et al. 2014)	F4063	MZUSP PL 636	–	Parque Estadual do Desengano, RJ	08/12/09	KF971679

## Results

### Molecular results

Tissue of the only *Ch. eudoxiae* Silva & Carbayo, sp. nov. individual was depleted during attempts to sequence it. COI sequences were obtained from the remaining ten specimens representing the three other new species, and *Ch. tristriata* and *Ch. albonigra*. Sequence alignments resulted in a matrix with 54 terminals and 679 base pairs. PartitionFinder indicated two partitions (first and second codon positions and third codon position), each with a different evolutionary model, with the TIM substitution model having gamma-distributed rate variations across sites and a proportion of invariable sites (TIM + I+ G) for the first and second codons, and a GTR substitution model with gamma-distributed rate variation across sites and a proportion of invariable sites (GTR + I + G) for the third codon partition.

**Figure 1.**
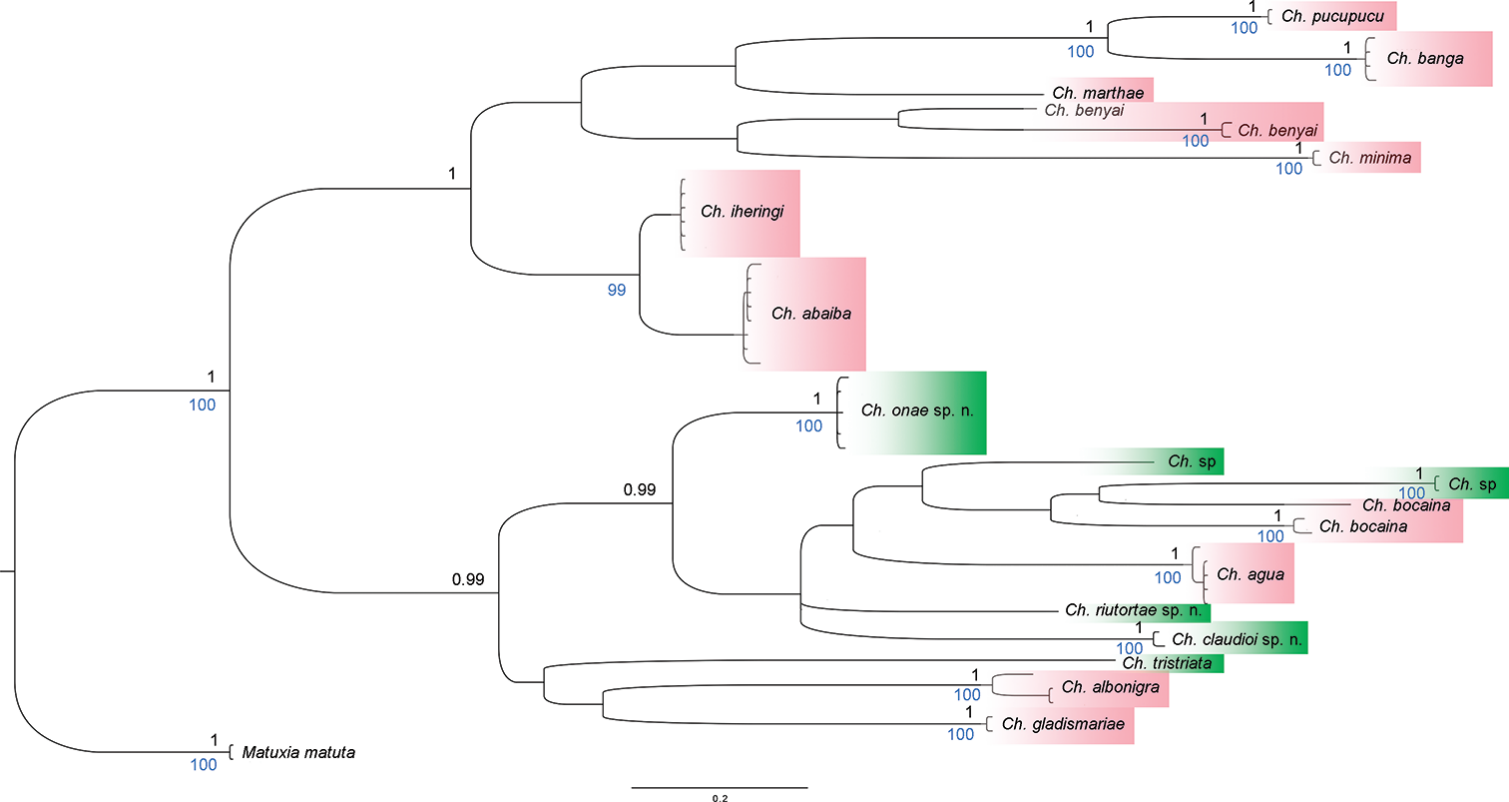
Phylogenetic tree of *Choeradoplana* obtained from the COI gene by Bayesian inference. Black numbers at the nodes correspond to the posterior probability (only values above 0.99 shown) and blue numbers to Bootstrap support in the tree by maximum likelihood (only values above 95% shown). See text for details.

Phylogenetic analyses under the two optimality criteria (BI and ML) retrieved the genus *Choeradoplana* and all morphological species as monophyletic with high posterior probabilities and bootstrap value, respectively (Fig. 1). Nonetheless, the internal morphology of MZUSP PL 1108 and MZUSP PL 2277 could not be assessed; these two specimens were nested in the clade of *Ch. onae* Lago-Barcia & Carbayo, sp. nov. The genus was split into two large clades in both trees composed of the same species, with high PP and bootstrap value. All species studied herein (with the exception of *Ch. eudoxiae* Silva & Carbayo, sp. nov.) are nested in one of these large clades, which includes species having an extrabulbar prostatic vesicle and lacking a penis papilla. However, independently from the optimality criteria applied, COI does not contain sufficient information to resolve the species interrelationships, thereby producing some polytomies and poorly supported clades.

### Taxonomic accounts

#### Family Geoplanidae Stimpson, 1857

**Subfamily Geoplaninae Stimpson, 1857**

***Choeradoplana* Graff, 1896**

##### Choeradoplana tristriata

Taxon classificationAnimaliaTricladidaGeoplanidae

(Schultze & Müller, 1857)

89394017-295F-55E1-A5D2-A439A06C941D

**Figure 2.**
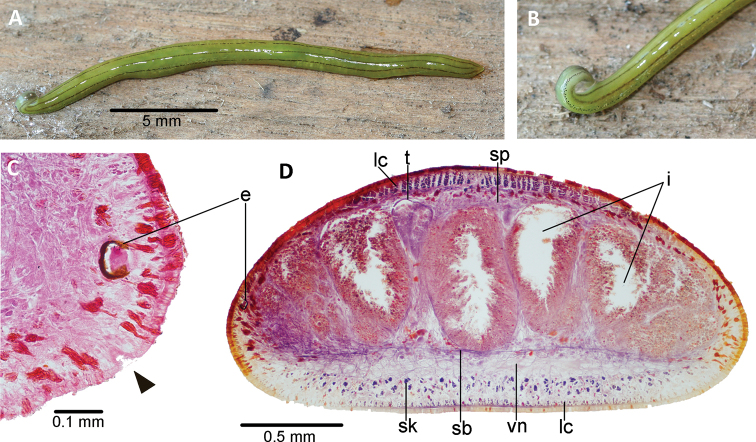
*Choeradoplana tristriata* (Schultze & Müller, 1857), specimen F3226 **A** dorsal view of the live specimen **B** lateral view of the anterior region of the live specimen **C** photomicrograph of a transverse section of the cephalic region, showing an eye and a sensory pit (arrowhead) **D** photomicrograph of a transverse section of the pre-pharyngeal region. Abbreviations: **cm** common muscle coat, **co** common glandular ovovitelline duct, **dd** decussate dorsal cutaneous muscles, **dm** diagonal decussate muscles, **e** eye, **ej** ejaculatory duct, **ep** esophagus, **er** erythrophil secretion, **fa** female atrium, **fd** female genital duct, **g** gonopore, **i** intestine, **lc** longitudinal cutaneous muscles, **ma** male genital atrium, **mk** Muskelgeflecht (Graff, 1899), **mo** mouth, **o** ovary, **ov** ovovitelline duct, **ph** pharyngeal pouch, **pp** penis papilla, **pv** prostatic vesicle, **px** pharynx, **rg** rhabditogen glands, **r** retractor muscle, **sb** subintestinal transverse muscles, **sd** sperm duct, **sg** shell glands, **sk** sunken longitudinal cutaneous muscles, **sm** spermatophore, **sn** subneural transverse muscles, **sp** supra-intestinal transverse muscles, **t** testis, **vi** vitellaria, **vn** ventral nerve plate.

**Figure 3.**
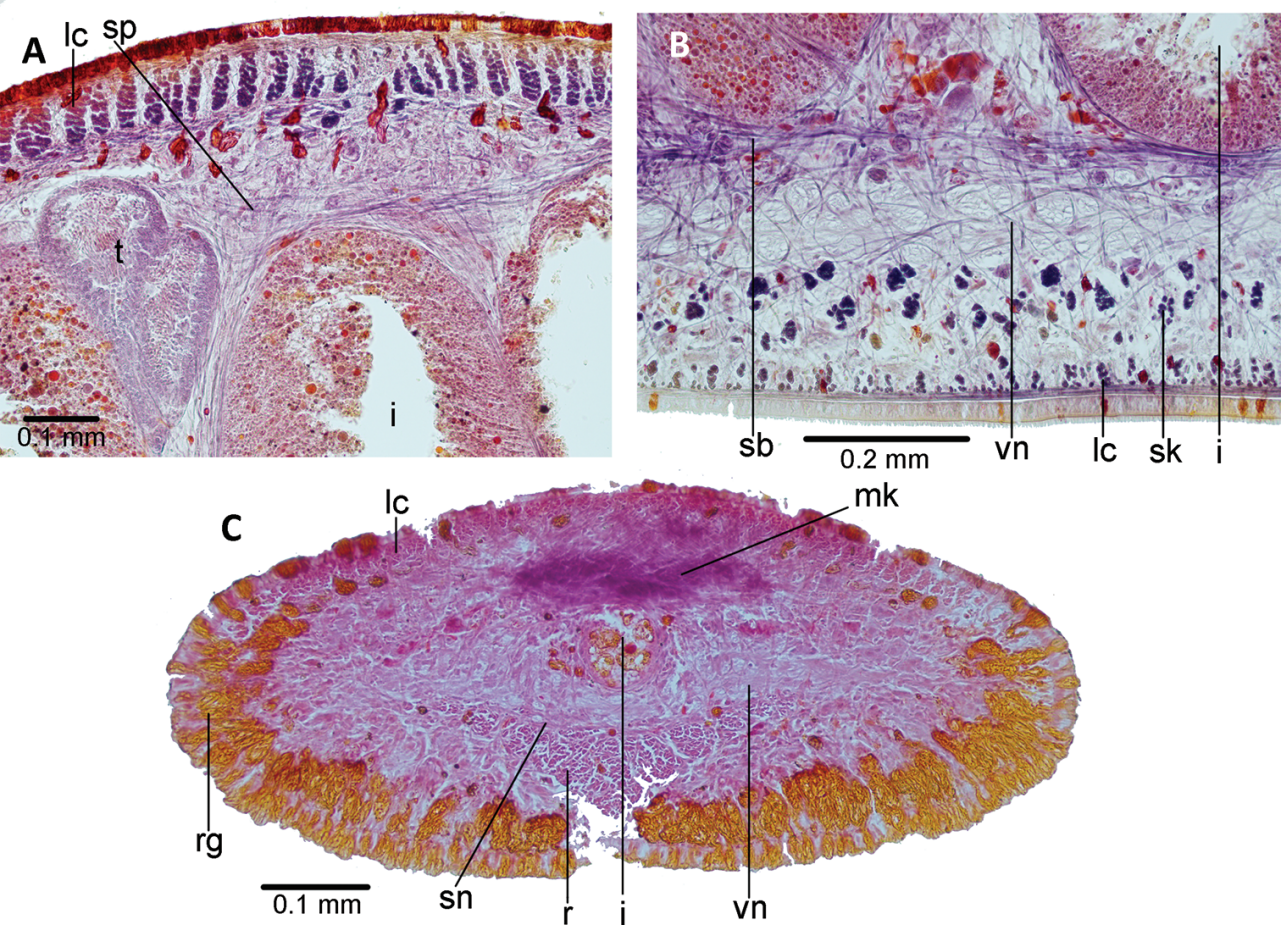
*Choeradoplana tristriata* (Schultze & Müller, 1857), specimen F3226. Photomicrographs of transverse sections **A** dorsal portion of the pre-pharyngeal region **B** ventral region of the pre-pharyngeal region **C** cephalic region. Abbreviations: **cm** common muscle coat, **co** common glandular ovovitelline duct, **dd** decussate dorsal cutaneous muscles, **dm** diagonal decussate muscles, **e** eye, **ej** ejaculatory duct, **ep** esophagus, **er** erythrophil secretion, **fa** female atrium, **fd** female genital duct, **g** gonopore, **i** intestine, **lc** longitudinal cutaneous muscles, **ma** male genital atrium, **mk** Muskelgeflecht (Graff, 1899), **mo** mouth, **o** ovary, **ov** ovovitelline duct, **ph** pharyngeal pouch, **pp** penis papilla, **pv** prostatic vesicle, **px** pharynx, **rg** rhabditogen glands, **r** retractor muscle, **sb** subintestinal transverse muscles, **sd** sperm duct, **sg** shell glands, **sk** sunken longitudinal cutaneous muscles, **sm** spermatophore, **sn** subneural transverse muscles, **sp** supra-intestinal transverse muscles, **t** testis, **vi** vitellaria, **vn** ventral nerve plate.

**Figure 4.**
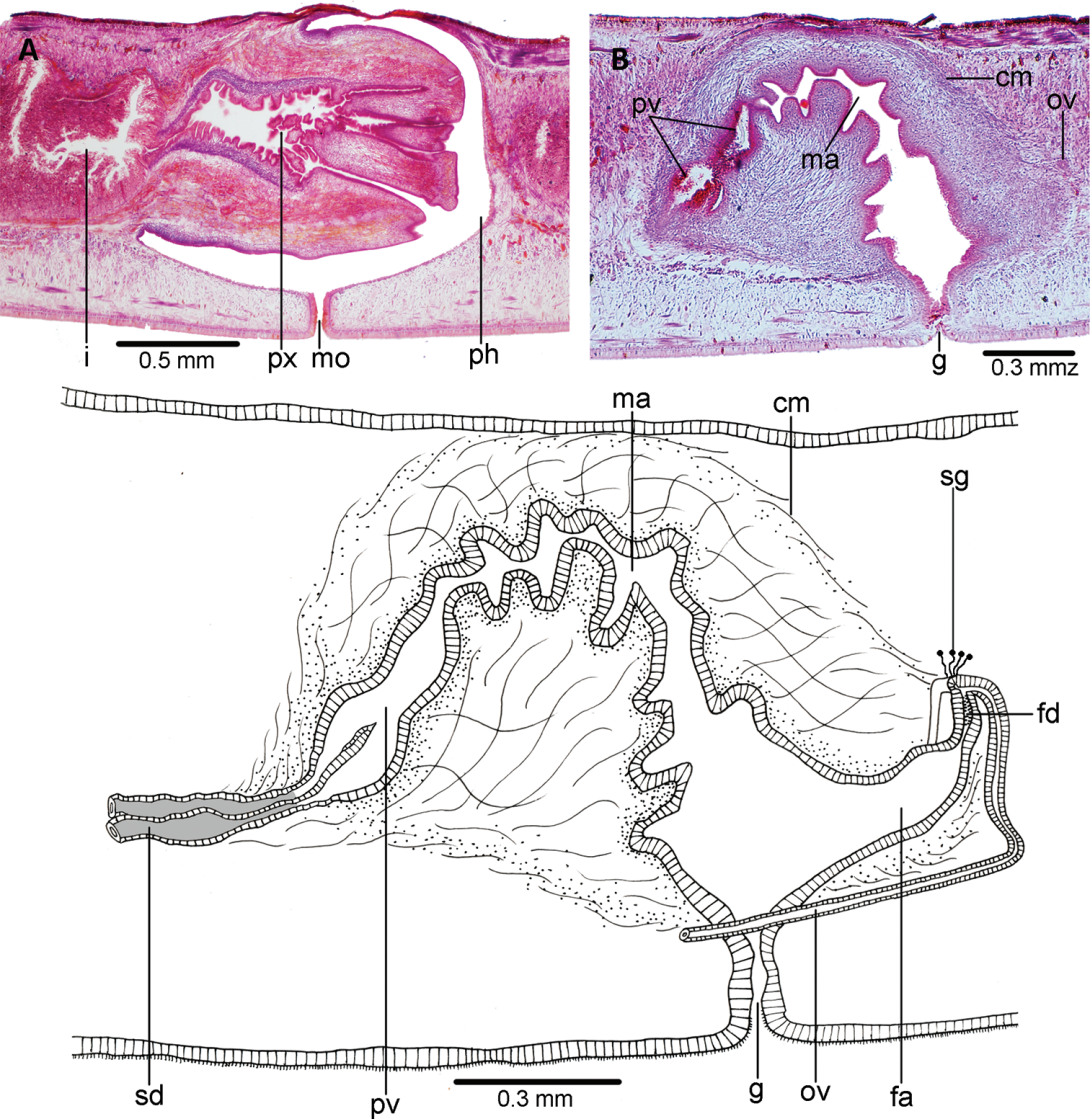
*Choeradoplana tristriata* (Schultze & Müller, 1857), specimen F3226 **A** photomicrograph of a sagittal section of the pharynx **B** photomicrograph of a sagittal section of the copulatory apparatus **C** diagrammatic representation of the copulatory apparatus. Abbreviations: **cm** common muscle coat, **co** common glandular ovovitelline duct, **dd** decussate dorsal cutaneous muscles, **dm** diagonal decussate muscles, **e** eye, **ej** ejaculatory duct, **ep** esophagus, **er** erythrophil secretion, **fa** female atrium, **fd** female genital duct, **g** gonopore, **i** intestine, **lc** longitudinal cutaneous muscles, **ma** male genital atrium, **mk** Muskelgeflecht (Graff, 1899), **mo** mouth, **o** ovary, **ov** ovovitelline duct, **ph** pharyngeal pouch, **pp** penis papilla, **pv** prostatic vesicle, **px** pharynx, **rg** rhabditogen glands, **r** retractor muscle, **sb** subintestinal transverse muscles, **sd** sperm duct, **sg** shell glands, **sk** sunken longitudinal cutaneous muscles, **sm** spermatophore, **sn** subneural transverse muscles, **sp** supra-intestinal transverse muscles, **t** testis, **vi** vitellaria, **vn** ventral nerve plate.

**Figure 5.**
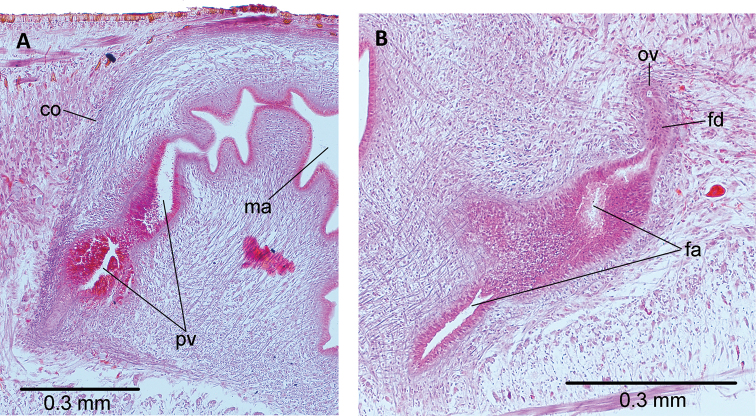
*Choeradoplana tristriata* (Schultze & Müller, 1857), specimen F3226. Photomicrographs of sagittal sections **A** prostatic vesicle and the male atrium **B** female atrium. Abbreviations: **cm** common muscle coat, **co** common glandular ovovitelline duct, **dd** decussate dorsal cutaneous muscles, **dm** diagonal decussate muscles, **e** eye, **ej** ejaculatory duct, **ep** esophagus, **er** erythrophil secretion, **fa** female atrium, **fd** female genital duct, **g** gonopore, **i** intestine, **lc** longitudinal cutaneous muscles, **ma** male genital atrium, **mk** Muskelgeflecht (Graff, 1899), **mo** mouth, **o** ovary, **ov** ovovitelline duct, **ph** pharyngeal pouch, **pp** penis papilla, **pv** prostatic vesicle, **px** pharynx, **rg** rhabditogen glands, **r** retractor muscle, **sb** subintestinal transverse muscles, **sd** sperm duct, **sg** shell glands, **sk** sunken longitudinal cutaneous muscles, **sm** spermatophore, **sn** subneural transverse muscles, **sp** supra-intestinal transverse muscles, **t** testis, **vi** vitellaria, **vn** ventral nerve plate.

Figures 1 , 2 , 3 , 4 , 5

Geoplana tristriata Schultze & Müller, 1857: 23.
Not Geoplana tristriata in Graff 1899: 327–328, 331, taf. V, figs 25, 26.
Pseudogeoplana tristriata : Ogren & Kawakatsu, 1990: 161.

###### Material examined.

MZUSP 2271 (field code, F3226), sexually mature: Parque Estadual da Serra de Tabuleiro, State of Santa Catarina, Brazil (-27.94, -48.79). coll. F. Carbayo and co-workers, 11 January 2009; transverse sections of the cephalic region on 6 slides; horizontal sections of the portion behind the cephalic extremity on 4 slides; transverse sections of the pre-pharyngeal region on 6 slides; sagittal sections of the pharynx and copulatory apparatus on 17 slides.

###### Distribution.

Municipality of Blumenau and Parque Estadual da Serra de Tabuleiro, State of Santa Catarina, Brazil.

###### Diagnosis.

*Choeradoplana* species with yellow green background color, with three thin discontinuous longitudinal black lines; ventral side is zinc-yellow. Copulatory apparatus compact, without penis papilla; female atrium funnel-shaped.

###### Description.

The preserved specimen measures 22 mm long and 2.5 mm wide (Fig. 2A). The body is slender and subcylindrical. The cephalic region is differentiated from the remaining body by means of a ‘neck’, laterally dilated and rolled up so that the ventral surface is provided with glandular cushions, and is facing out (Fig. 2B); the posterior extremity is pointed. The creeping sole is 90% of body width at the pre-pharyngeal region. The mouth is positioned at a distance from the anterior extremity equal to 60% of the body length, and the gonopore is at 74%.

The dorsal coloration consists of a yellow green (RAL 1018) background color, with three thin discontinuous longitudinal lines of small black spots. These spots are less concentrated in the median line (Fig. 2A). The ventral side is zinc yellow (RAL 1018), except for a silver grey (RAL 7001) spot on the glandular cushions (Fig. 2A).

The eyes are formed by one pigmented cup of 80 μm in diameter (Fig. 2C). There are no clear halos around them. Eyes are absent in the very anterior extremity of the body; at 1.5 mm behind the anterior tip the eyes are marginally distributed in a row of two or three eyes; 3.8 mm posterior to the anterior tip, the eyes are placed in a single marginal row which runs along the whole body until posterior extremity, with each eye at a distance of ~ 0.3 mm from each other (Fig. 2B).

Sensory pits are 25 μm deep (Fig. 2C, arrowhead), and are distributed ventro-laterally in a uniserial row between ~ 0.4 mm behind the anterior extremity to at least the ovarian level.

Numerous rhabditogen cells discharge through the glandular cushions of the cephalic region. These cells are scarce in the dorsal epidermis. Abundant erythrophil gland cells pierce the dorsal and marginal epithelium in the pre-pharyngeal region. The entire epithelium is additionally pierced by scarce gland cells of two types, producing cyanophil and xanthophil granules, respectively. No glandular margin.

The cutaneous musculature of the pre-pharyngeal region comprises a subepithelial circular muscle, followed by a diagonal layer with decussate fibers, and a longitudinal muscle organized in tight bundles (Figs 2D, 3A). This longitudinal muscle is 70 μm thick dorsally; it is ventrally divided into a 35 μm-thick muscle, organized in bundles with 4–8 fibers each, and a 50 μm-thick muscle sunken into the parenchyma consisting of scattered bundles with 5–12 fibers each (Fig. 3B). The thickness of the cutaneous muscle coat is 17% of the body height.

There are three parenchymal muscle layers in the pre-pharyngeal region (Fig. 3A, B): a well-developed dorsal layer of diagonal, decussate fibers (12–18 μm thick); a transverse supra-intestinal muscle (22–25 μm); and a transverse subintestinal muscle (30–35 μm).

The ventral longitudinal cutaneous muscle is modified into the retractor muscle in the cephalic region. This retractor muscle is delta-shaped in cross-section along 0.7 mm (or 3% of body length), starting 0.2 mm (or 1%) from the anterior extremity of the body (Fig. 3C), and its thickness is 24% of the height of the cephalic region. The dorsal decussate and subintestinal parenchymal muscles in this region are weak, whereas the supra-intestinal is strongly developed and mixed with dorso-ventral muscle fibers giving rise to the Muskelgeflecht or interwoven muscle, and is 75 μm in thickness. A fourth subneural parenchymal muscle is present in the cephalic region, and is located beneath the central nerve system and above the retractor muscle. The paired glandular cushions are pierced by numerous rhabditogen cells. The arrangement of cutaneous and parenchymal muscles in the cephalic region and the glandular component of the cephalic cushions match those of the type species of the genus, *Choeradoplana iheringi* Graff, 1899.

The mouth is located in the middle of the pharyngeal pouch (Fig. 4A). It is a bell-shaped pharynx, with the dorsal insertion posterior to the ventral insertion ~ 45% of the pharyngeal length. An esophagus is not present. The pharyngeal pouch is lined with a non-ciliated, cuboidal-to-flat epithelium, underlain by a one-fiber thick layer of longitudinal muscle, followed by a 10 μm-thick layer of circular muscle. The outer pharyngeal epithelium is cuboidal, ciliated, underlain by a longitudinal muscle (7.5 μm thick), followed by a circular muscle (45 μm thick) with interspersed longitudinal fibers; the inner epithelium is flat, ciliated, underlain by a circular muscle (62 μm thick) with interspersed longitudinal fibers. Numerous erythrophil and xanthophil gland cells open and discharge their contents through the distal portion of the pharynx.

The testes are dorsal, located between the intestinal diverticula, with some of them reaching the parenchymal supra-intestinal transverse muscle (Figs 2D, 3A). The testes are arranged in two paramedian rows. They extend from 1 mm behind the level of the ovaries (30% of body length) to 1.2 mm from the root of the pharynx. Sperm ducts run straight and immediately above the subintestinal parenchymatic muscle. They distally penetrate the anterior region of the common muscle coat (Fig. 4B, C) to open into the proximal portion of the paired branches of the prostatic vesicle. The prostatic vesicle consists of a proximal half of these paired branches and a distal unpaired half. This vesicle runs postero-dorsally to open into the dorso-anterior section of the male atrium. An ejaculatory duct and penis papilla are absent (Figs 4C, 5A). The penis bulb is thick and consists of numerous muscle fibers continuous with those underlying the epithelium of the male atrium. The prostatic vesicle is lined with a columnar-to-cuboidal epithelium, underlain by a thin longitudinal muscle (5 μm thick), followed by a 30 μm-thick circular muscle interspersed with longitudinal fibers. The paired portion of the prostatic vesicle receives abundant erythrophil and xanthophil granules from the respective gland cells, while the unpaired portion receives abundant cyanophil and xanthophil granules.

The male atrium is long and narrow with folded walls. The proximal third of the atrium runs postero-dorsally; distal two-thirds runs ventrally almost vertically above the gonopore canal. The atrium is lined by a cuboidal-non-ciliated epithelium, and underlain by an 80 μm-thick circular muscle with interspersed longitudinal fibers. The male atrium is 1.2× longer than the female atrium.

The ovaries are mature, ovoid, and 200 μm in length. They are located above the ventral nerve plate at a distance from the anterior body tip equal to 25% of its body length (5.5 mm from anterior tip). Ovovitelline ducts emerge from the dorso-lateral aspect of the ovaries, where-after they run backwards above the ventral nerve plate. The ducts bend medially, posteriorly to the female atrium, and subsequently ascend vertically and medially to communicate with each other above the postero-dorsal section of the female atrium (Fig. 5B). The ovovitelline ducts open directly into a very short female genital canal lined by a cuboidal ciliated epithelium. A small number of small shell gland cells can be spotted around the junction of the two ovovitelline ducts.

The female atrium is funnel-shaped, not folded, and lined by a ciliated columnar-to-cuboidal epithelium, which is surrounded by circular muscle fibers with interspersed longitudinal fibers. This muscle is continuous with the common muscle coat. Most of the abundant gland cells discharging into the female atrium have a fine granular xanthophil and erythrophil secretion. The length:height ratio of the copulatory apparatus enveloped by the common muscle coat is 1.4:1.

###### Remarks.

**On the identity of our specimen.**Schultze and Müller (1857) described *Geoplana tristriata* from near Blumenau, in the State of Santa Catarina. The original description reads: “*Geoplana tristriata*, pale yellowish-green, with three narrow, dark, longitudinal lines on the back; belly paler. Greatest breadth at approx. the second third part of the length, where the mouth is situated. It likes to bend the head upwards. At the point of curvature on each side there is a closed packed group of eye-spots, which are continued in an irregular series to the posterior extremity. The anterior margin of the head appears to be destitute of eyes. Length 1 1/2 inch [38.1 mm]; breadth 1 1/2 line [3.81 mm]. Abundant.”

There is no record of the deposition of the type series. It was probably not preserved. Our specimen was collected 115 km south from the type locality, and matches Schultze and Müller’s species in all characteristics. The conspecificity of our specimen could be questioned since the copulatory apparatus is the most important organ assuring identification in triclads. However, this species combines a set of unusual features among Geoplanins: the color pattern of the body, the cephalic region bent to the dorsal side (actually only found in *Choeradoplana* and *Cephaloflexa* among Geoplaninae), and the absence of eyes in the very anterior tip of the body. Schultze and Müller’s and our specimen sharing these uncommon attributes supports their conspecificity.

The species redescribed herein also matches all diagnostic characteristics of *Choeradoplana* and should therefore be transferred to this genus. The species is unique in the external aspect in that there are no other species of *Choeradoplana* with dorsal green color with three longitudinal dark stripes. Internally, *Choeradoplana bilix* Marcus, 1951; *Ch. crassiphalla* Negrete & Brusa, 2012; *Ch. langi* (Dendy, 1894); and *Ch. marthae* Froehlich, 1955 are similar to *Ch. tristriata* in the compact aspect of the copulatory apparatus, having a length:height ratio equivalent to 1.8:1 or less, as calculated in drawings (du Bois-Reymond Marcus 1951; Marcus 1951; Froehlich 1959; Negrete and Brusa 2012). However, *Ch. bilix*, *Ch. crassiphalla* and *Ch. marthae* possess a penis papilla (vs. absent in *Ch. tristriata*), and the female atrium of *Ch. langi* is a narrow canal (vs. a funnel-shaped cavity).

**The identity of Graff’s specimen and subsequent taxonomic actions.** Herman von Ihering collected one specimen in Taquara, State of Rio Grande do Sul, Brazil, which he identified as a member of this species. Ihering sent it to Graff (1899), who endorsed his identification, but only provided a description of the external aspect. Froehlich (1959) disagreed with Ihering’s identification because of the body shape, the relative position of the mouth, and the different width of the paramedian dorsal stripes. Froehlich concluded that both Schultze and Müller’s and Graff’s species remain obscure (Froehlich 1959), but did not propose taxonomic changes. Ogren and Kawakatsu (1990) considered Schultze and Müller’s and Graff’s specimens conspecific, and transferred the species to the *Pseudogeoplana* collective genus, which houses species lacking information about the internal organs, especially the copulatory apparatus. We agree with Froehlich’s opinion that Graff’s species is different from Schultze and Müller’s species. Accordingly, we propose the name *Pseudogeoplana aevipandemiae* Lago-Barcia & Carbayo, sp. nov. for Graff’s species. The specific epithet means ‘from the times of the pandemic’. The epithet alludes to the COVID-19 pandemic and is intended to keep the memory of the negative effects caused by the long months of closure of the laboratories for the conclusion of this paper.

We did not find Graff’s specimen in the museums where part of this collection was disseminated (Naturhistorisches Museum, Basel; Museum of Natural History, Vienna; Senckenberg Museum, Frankfurt; Zoological Museum, Hamburg; Natural History Museum, London). Therefore, we consider Graff’s specimen lost.

##### Choeradoplana albonigra

Taxon classificationAnimaliaTricladidaGeoplanidae

(Riester, 1938)

47E025EE-24A4-516F-A104-6F99A7FDC91E

**Figure 6.**
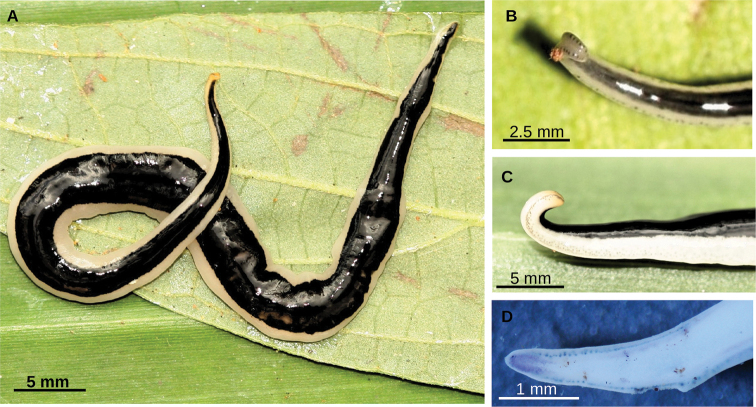
*Choeradoplana albonigra* (Riester, 1938) **A** habitus of living specimen F4024 in dorsal view **B** detail of anterior extremity of living specimen F2313 in dorsal view. Debris is glued on the body **C** detail of anterior extremity of living specimen F4024 in lateral view **D** detail of anterior extremity of preserved specimen F4031 in ventral view. Abbreviations: **cm** common muscle coat, **co** common glandular ovovitelline duct, **dd** decussate dorsal cutaneous muscles, **dm** diagonal decussate muscles, **e** eye, **ej** ejaculatory duct, **ep** esophagus, **er** erythrophil secretion, **fa** female atrium, **fd** female genital duct, **g** gonopore, **i** intestine, **lc** longitudinal cutaneous muscles, **ma** male genital atrium, **mk** Muskelgeflecht (Graff, 1899), **mo** mouth, **o** ovary, **ov** ovovitelline duct, **ph** pharyngeal pouch, **pp** penis papilla, **pv** prostatic vesicle, **px** pharynx, **rg** rhabditogen glands, **r** retractor muscle, **sb** subintestinal transverse muscles, **sd** sperm duct, **sg** shell glands, **sk** sunken longitudinal cutaneous muscles, **sm** spermatophore, **sn** subneural transverse muscles, **sp** supra-intestinal transverse muscles, **t** testis, **vi** vitellaria, **vn** ventral nerve plate.

**Figure 7.**
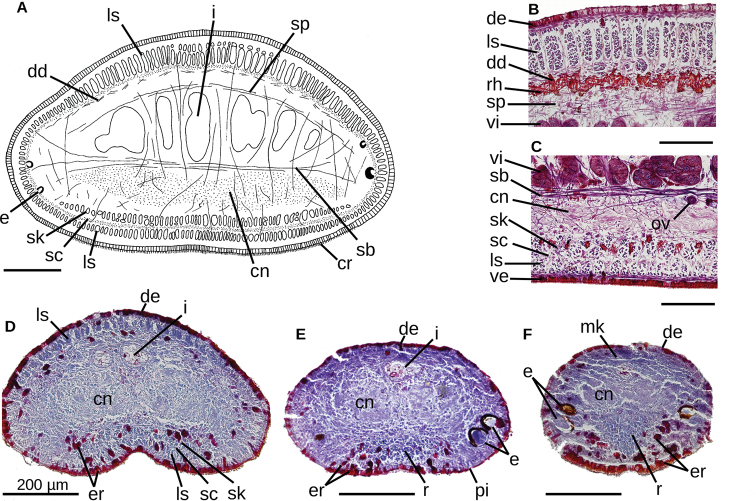
*Choeradoplana albonigra* (Riester, 1938) **A** diagrammatic transverse section of a portion, seemingly near the anterior extremity, of holotype. Photomicrographs of transverse sections (**B–F**) **B** dorsal epidermis in pre-pharyngeal region of specimen F4024 **C** ventral epidermis in pre-pharyngeal region of specimen F4024 **D–F** cephalic region of specimen F4081 at 1.2, 0.4, and 0.15 millimeters from anterior extremity of the body, respectively. Scale bar: 200 µm. Abbreviations: **cm** common muscle coat, **co** common glandular ovovitelline duct, **dd** decussate dorsal cutaneous muscles, **dm** diagonal decussate muscles, **e** eye, **ej** ejaculatory duct, **ep** esophagus, **er** erythrophil secretion, **fa** female atrium, **fd** female genital duct, **g** gonopore, **i** intestine, **lc** longitudinal cutaneous muscles, **ma** male genital atrium, **mk** Muskelgeflecht (Graff, 1899), **mo** mouth, **o** ovary, **ov** ovovitelline duct, **ph** pharyngeal pouch, **pp** penis papilla, **pv** prostatic vesicle, **px** pharynx, **rg** rhabditogen glands, **r** retractor muscle, **sb** subintestinal transverse muscles, **sd** sperm duct, **sg** shell glands, **sk** sunken longitudinal cutaneous muscles, **sm** spermatophore, **sn** subneural transverse muscles, **sp** supra-intestinal transverse muscles, **t** testis, **vi** vitellaria, **vn** ventral nerve plate.

**Figure 8.**
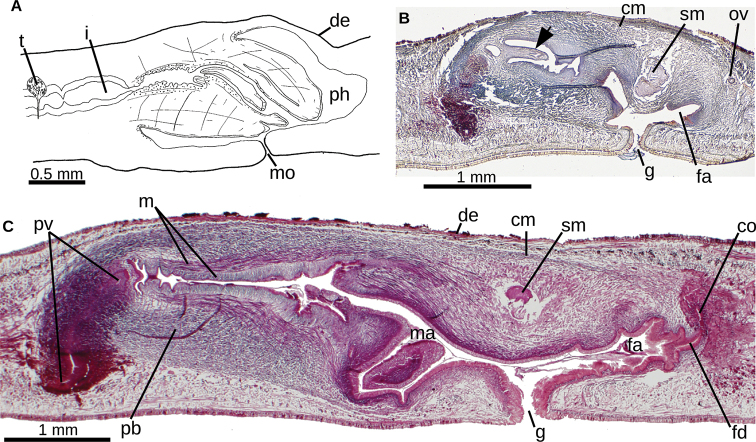
*Choeradoplana albonigra* (Riester, 1938) **A** diagrammatic representation of the pharynx from sagittal sections of holotype **B** sagittal section of the copulatory apparatus of specimen F2391; note the small finger-shaped fold of the male atrium (arrow) **C** photomicrograph of a sagittal section of the copulatory apparatus of specimen F4024. Abbreviations: **cm** common muscle coat, **co** common glandular ovovitelline duct, **dd** decussate dorsal cutaneous muscles, **dm** diagonal decussate muscles, **e** eye, **ej** ejaculatory duct, **ep** esophagus, **er** erythrophil secretion, **fa** female atrium, **fd** female genital duct, **g** gonopore, **i** intestine, **lc** longitudinal cutaneous muscles, **ma** male genital atrium, **mk** Muskelgeflecht (Graff, 1899), **mo** mouth, **o** ovary, **ov** ovovitelline duct, **ph** pharyngeal pouch, **pp** penis papilla, **pv** prostatic vesicle, **px** pharynx, **rg** rhabditogen glands, **r** retractor muscle, **sb** subintestinal transverse muscles, **sd** sperm duct, **sg** shell glands, **sk** sunken longitudinal cutaneous muscles, **sm** spermatophore, **sn** subneural transverse muscles, **sp** supra-intestinal transverse muscles, **t** testis, **vi** vitellaria, **vn** ventral nerve plate.

**Figure 9.**
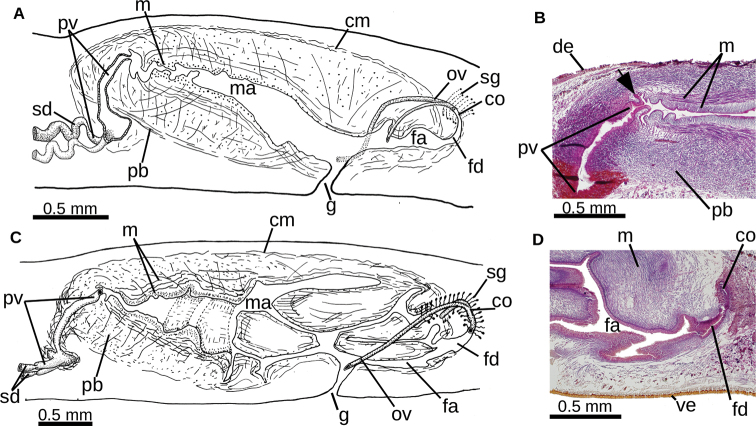
*Choeradoplana albonigra* (Riester, 1938) **A** diagrammatic reconstruction of copulatory apparatus of holotype from sagittal sections **B** photomicrograph of a sagittal section of the unpaired portion of the prostatic vesicle and proximal section of male atrium of specimen F4024. Arrow points the small ring-shaped horizontal fold communicating prostatic vesicle with male atrium **C** diagrammatic reconstruction of copulatory apparatus of specimen F4081 from sagittal sections **D** photomicrograph of a sagittal section of the female atrium of specimen F4081. Abbreviations: **cm** common muscle coat, **co** common glandular ovovitelline duct, **dd** decussate dorsal cutaneous muscles, **dm** diagonal decussate muscles, **e** eye, **ej** ejaculatory duct, **ep** esophagus, **er** erythrophil secretion, **fa** female atrium, **fd** female genital duct, **g** gonopore, **i** intestine, **lc** longitudinal cutaneous muscles, **ma** male genital atrium, **mk** Muskelgeflecht (Graff, 1899), **mo** mouth, **o** ovary, **ov** ovovitelline duct, **ph** pharyngeal pouch, **pp** penis papilla, **pv** prostatic vesicle, **px** pharynx, **rg** rhabditogen glands, **r** retractor muscle, **sb** subintestinal transverse muscles, **sd** sperm duct, **sg** shell glands, **sk** sunken longitudinal cutaneous muscles, **sm** spermatophore, **sn** subneural transverse muscles, **sp** supra-intestinal transverse muscles, **t** testis, **vi** vitellaria, **vn** ventral nerve plate.

Figures 6 , 7 , 8 , 9

Geoplana albonigra Riester, 1938: 7–9, figs 4, 5, 86, 87, taf. 1, fig. 2.
Notogynaphallia albonigra : Ogren & Kawakatsu, 1990: 140.
Choeradoplana albonigra : Carbayo et al., 2013: 514, 517.

###### Material examined.

***Holotype***SMF N° 702, sexually mature: Teresópolis, Rio de Janeiro, Brazil, coll. E. Bresslau, April 9^th^, 1914; transverse sections of a body portion seemingly being close to the anterior tip of the body on 1 slide; sagittal sections of the pharynx and copulatory apparatus on 1 slide; anterior and posterior tips in Canada balsam on 1 slide.

Reserva Biológica Augusto Ruschi, municipality of Santa Teresa, State of Espírito Santo, Brazil. May 26–27^th^, 2008. **MZUSP PL 1109** (field code, F2313), only anterior half of the body collected: transverse sections of anterior extremity on 10 slides; sagittal sections of a portion behind anterior extremity on 5 slides; horizontal sections containing testes on 32 slides. **MZUSP PL 1113** (field code, F2391), sexually mature: transverse sections of anterior extremity on 9 slides; sagittal sections of ovaries and testes on 6 slides; sagittal sections of pharynx and copulatory apparatus on 11 slides.

Parque Estadual do Desengano, municipality of Santa Maria Madalena, State of Rio de Janeiro, Brazil. August 10–13^th^, 2009. **MZUSP PL 2273** (field code, F3991), juvenile: horizontal sections of anterior extremity on 3 slides. **MZUSP PL 2153** (field code, F4031), sexually mature: transverse sections of anterior extremity on 12 slides; horizontal sections of ovaries on 22 slides; transverse sections of pre-pharyngeal region on 4 slides; sagittal sections of pharynx and copulatory apparatus on 8 slides; horizontal sections of posterior extremity on 4 slides. **MZUSP PL 2152** (field code, F4024), sexually mature: horizontal sections of anterior extremity on 7 slides; sagittal sections of ovaries on 19 slides; horizontal sections of testes on 21 slides; transverse sections of pre-pharyngeal region on 9 slides; sagittal sections of pre-pharyngeal region on 12 slides; sagittal sections of copulatory apparatus on 12 slides. **MZUSP PL 1083** (field code, F4081), sexually mature: transverse sections of anterior extremity on 55 slides; sagittal sections of the ovaries on 25 slides; horizontal sections of testes on 9 slides; transverse sections of pre-pharyngeal region on 6 slides; sagittal sections of pharynx and copulatory apparatus on 38 slides; horizontal sections of posterior extremity on 24 slides.

###### Distribution.

Reserva Biológica Augusto Ruschi, Santa Teresa, State of Espírito Santo; Parque Estadual do Desengano, Santa Maria Madalena, State of Rio de Janeiro; Municipality of Teresópolis, State of Rio de Janeiro, Brazil.

###### Diagnosis.

*Choeradoplana* species with a white dorsum, covered by a wide median black band, darker at its margins; an additional thin black median stripe may be present. The anterior third of the body is progressively thinner towards the pointed tip; its extremity has no lateral dilations or “neck” differentiating the head from the body. The ventral side of the cephalic region is concave and without glandular cushions. The proximal third of the prostatic vesicle is extrabulbar. The copulatory apparatus is relatively long; penis papilla is absent, and the female atrium is approximately funnel-shaped.

###### Description.

Living specimens range between 50–63 mm in length and 2–3 mm in width (n = 2). Preserved specimens range between 45–73 mm in length and 2–4 mm in width (n = 4). The body is slender and subcylindrical, with the anterior third becoming progressively thinner to the anterior tip. The anterior extremity is very thin and coiled up so that the ventral surface is facing out (Fig. 6A, B). The ventral side of the cephalic region is slightly concave with indistinct glandular cushions (Fig. 6B–D). The posterior extremity is pointed. The creeping sole is 80–86% of width in the pre-pharyngeal region (n = 3) (Fig. 7A). Its mouth is at a distance from the anterior extremity ranging between 50.9–64.4% of body length, gonopore at 63.2–75.3% (n = 3).

The background color of the body is traffic white (RAL 9016). It is dorsally covered by a wide graphite black (RAL 9011) band, darker at its margins, as wide as three-quarters of the body width, in the middle of which runs a thin jet black (RAL 9005) stripe which is not apparent in some individuals. Anterior and posterior extremities of the body are slightly orangish. The color has faded out in preserved specimens. The dorsal graphite black band in the body tips of the holotype split into two brownish stripes.

The eyes are of one-pigment cup type, 25–45 µm in diameter; with no clear halos. They are marginally distributed in an irregular row of 2–6 eyes, from the anterior tip of the body (Figs 6B, C, 7A), backwards to the posterior end. Anterior extremity devoid of eyes.

The sensory pits are 22–27 μm deep and ventro-laterally are distributed in a single row along approximately the anterior one-seventh of the body. The pits are absent at the very anterior tip of the body (300 µm).

Numerous rhabditogen cells open onto the dorsal surface of the body (Fig. 7B) in the pre-pharyngeal region and its margins; these margins are also pierced by scarce gland cells producing granular, erythrophil secretion. The ventral epithelium is pierced by scarce gland cells producing granular, xanthophil secretion, and abundant gland cells producing strong erythrophil secretion (Fig. 7C). There is no glandular margin.

The cutaneous musculature consists of a subepithelial circular muscle, followed by a diagonal layer with decussate fibers, and a strong longitudinal muscle organized in bundles (Fig. 7A–C). This longitudinal muscle is 81.2–175 μm thick dorsally; it is ventrally divided into a 37.5–50.0 μm-thick muscle, organized in bundles with 20–35 fibers each, and an equally thick muscle sunken into the parenchyma, and constituted by scattered bundles with 7–30 fibers each (Fig. 7B). A few dorsal longitudinal fibers are medially intermingled with those of the parenchymatic dorsal layer of diagonal decussate fibers. The thickness of the cutaneous muscle coat is 22–25% (n = 3) of the body height.

The pre-pharyngeal region, namely the dorsal decussate muscle (40–55 μm thick, n = 2), transverse supra-intestinal muscle (74–100 μm); and transverse subintestinal muscle (45–65 μm) (n = 2) (Fig. 7A–C).

The retractor muscle of the head is delta-shaped in a cross-section along 0.3 mm (or 0.5% of body length) from behind, 0.1 mm (or 0.15%) of the anterior extremity of the body (Fig. 7C), and its thickness equals 36% of the height of the cephalic region. The Muskelgeflecht is 18–25 μm thick (24% of body height). The subneural parenchymal muscle consists of a few scarce transverse fibers. The glandular cushions are composed of a relatively small quantity of rhabditogen cells (Fig. 7D–F).

The mouth is located at a distance from the anterior section of the pharyngeal pouch ranging between 55.1–78.9% (n = 4) (Fig. 8A). The pharynx is bell-shaped with dorsal insertion near the mouth level. An esophagus is absent. The outer epithelium of the pharynx is cuboidal, ciliated and underlain by a thin longitudinal muscle (3–5 μm); followed by a circular muscle (15–20 μm) with interspersed longitudinal fibers. The inner pharyngeal epithelium is ciliated, underlain by a circular muscle (50 µm thick; n = 3) with longitudinal fibers interspersed. There is abundant granular secretion of three types, cyanophil, erythrophil, and xanthophil, respectively, and pierce the distal pharyngeal epithelium.

The testes are dorsal, located under the supra-intestinal transverse muscle layer, partially placed between the intestinal diverticula. They extend from the level of the ovaries (a distance from the anterior extremity of the body equal to 27% of the body length) to nearly the root of pharynx (53% of the body length). Sperm ducts run immediately above the subintestinal muscle layer, dorsally and slightly laterally to the ovovitelline ducts. Distal portions of sperm ducts contain sperm, and are surrounded by a circular muscle. Sperm ducts communicate with the two roughly horizontal branches of the extrabulbar portion of the prostatic vesicle. These branches open laterally into an irregular, pear-shaped cavity, which is located more or less under the anterior section of the penis bulb (Figs 8B, C, 9A–C). The prostatic vesicle continues as an almost vertical, tubular portion inside the very dense penis bulb to bend posteriorly towards a loose small ring-shaped horizontal fold (or ‘small penis-shaped fold’, after Riester 1938) which may be narrowed and elongated as a finger (Fig. 8B, arrow). This fold gives passage to the male atrium. The penis bulb is very thick and consists of very numerous muscle fibers which are continuous with those underlying the epithelium of the male atrium. There is no ejaculatory duct as a differentiated portion. The prostatic vesicle is lined with a columnar, ciliated epithelium. The very abundant secretions discharging into it are zoned along the prostatic vesicle: paired branches receive very fine granular erythrophil and pink-reddish staining secretion; the dilated portion takes gross granular erythrophil intensely reddish staining secretion; a proximal intrabulbar portion receives finely erythrophil secretion; the distal section takes both finely cyanophil granular secretion and xanthophil variously sized secretion granules (Fig. 9B). The extrabulbar portion is surrounded by interwoven muscle fibers, and the intrabulbar portion by a muscular layer of circular fibers interspersed with longitudinal ones, both portions are 20–35 μm thick. The loose small ring-shaped horizontal fold is lined with a 3–6 μm high non-ciliated epithelium and is surrounded by a few seemingly circular muscle fibers.

The proximal half of the male atrium is slightly folded and narrow. The distal half is ample and is narrowed distally by a large, dorsal fold extending through both the male and female atria. The stroma of this fold is strongly muscularized with longitudinal and oblique fibers. Additional lateral folds may be present in the distal half (Figs 8B, C, 9B).

The proximal half of the male atrium is lined with a 5 μm high non-ciliated, infra-nucleated epithelium which is pierced by scarce gland cells producing fine erythrophil granules, and by gland cells producing variously sized xanthophil granules. This epithelium is surrounded by a 20–60 μm-thick dense muscle of very thin muscle fibers (2 μm); followed by a muscle (< 150 μm thick) of 4 μm-thick fibers. The distal half of the male atrium is lined with an epithelium which is pierced by gland cells producing erythrophil granules. An intensely reddish erythrophil is found beneath this epithelium secretion. The lining epithelium of the distal half of the male atrium is surrounded by a circular muscle (18–45 μm); followed by a longitudinal muscle (80–150 μm) (n = 3).

A spermatophore is in the stroma of the large dorsal fold leveled with the gonopore in three specimens (F2391, F4024, F4081) (Fig. 8B, C). The spermatophore is ovoid in shape, with approximately 150 μm in diameter, and seems to be constituted of a central mass of sperm partially surrounded by irregular strands of erythrophil granules. The biological meaning of the position of the spermatophore will be discussed elsewhere.

The ovaries are mature, very elongated, and club-shaped due to the dilated proximal extremity. The thin portion can be divided into smaller segments. The size of the ovaries ranges between 700–1200 µm in length and 120–170 µm in width (n = 4). They are located above the ventral nerve plate, and at a distance from the anterior extremity equal to 27.1–31.3% of the body length (n = 2). Ovovitelline ducts emerge from the dorso-lateral aspect of the ovaries and run above the nerve plate. The proximal segment of the oviducts is dilated and contains sperm (n = 4). They ascend posteriorly and medially laterally to the female atrium, then unite dorsally to the common glandular ovovitelline duct (Fig. 9A–C). The distal portion of the oviducts is pierced by shell gland cells. The common glandular ovovitelline duct is downwards directed; it is continuous with a posteriorly and upward directed diverticulum of the female atrium, i.e., the female genital canal. The female atrium is roughly funnel-shaped, narrowed by a large dorsal fold continued with that of the male atrium. The female genital duct is lined with epithelial cells, with the apical portion containing fine erythrophil granules, and is surrounded by a thin layer of circular and longitudinal muscle fibers.

The female atrium is lined with a columnar, non-ciliated epithelium, and is pierced by two types of cells producing erythrophil and cyanophil granular secretions, respectively. The female atrium is as long as half the male atrium. The common muscular coat of the copulatory apparatus is composed of a weak layer of intermingled fibers; it is 20–40 μm thick in the anterior section, and thinner in the posterior section. The length:height ratio of the copulatory apparatus enveloped by the common muscle coat ranges between 3.0–3.5:1 (n = 5; mean 3.2:1).

###### Behavioral note.

Creeping on the Petri dish, the animals sometimes rise up to three-quarters of the body from the substrate and swing to the sides as they would be searching for ground. When touched, they can react by tumbling. A similar behavior, named ‘escape reaction’, was observed for *Ch. marthae* by Froehlich (1959).

###### Remarks.

This species was originally described as *Geoplana albonigra* Riester, 1938, from Teresópolis, State of Rio de Janeiro, Brazil. The external aspect and the internal morphology of the pharynx and copulatory apparatus were given in the original description. The species was later transferred to the recently proposed new genus *Notogynaphallia* Ogren & Kawakatsu, 1990, erected for species of Geoplaninae without penis papilla and dorsally located female genital canal. Based on morphological and molecular information, Carbayo et al. (2013) transferred the species to *Choeradoplana*, but they did not provide morphological evidence supporting that taxonomic decision.

**On the identity of our specimens.** Our specimens and the holotype of *Ch. albonigra* are much alike, with the exception of the size of the male and female atria. Whereas the male and female atria are relatively narrow in the holotype, these atria are higher in two out of four mature individuals. Differences of the same nature have been observed in other land planarians (e.g., *Pasipha pasipha* (Marcus, 1951); *Obama josefi* (Carbayo & Leal-Zanchet, 2001)), and this might be caused by different states of maturation of each specimen, albeit all being mature, or by the physiological state of the individuals such as a recent copulation, as seems to be the case of the specimens bearing a spermatophore.

**The systematic position of Ch. albonigra.** Riester described the species from a single individual and assigned the species to *Geoplana*. In Riester’s description, there is no reference to the inner structures of the cephalic region, nor to the cutaneous muscle coat. The cephalic region of the holotype is embedded in Canada balsam on a histological glass slide. Our study revealed a set of additional aspects, including the delta-shaped retractor muscle of the head and the in-sunk ventral longitudinal cutaneous muscle which only agree with *Choeradoplana*. Our molecular data also support this conclusion. However, *Ch. albonigra* deviates from the genus in the body shape, since its anterior third narrows progressively towards the anterior extremity, with the latter also lacking the typical glandular cushions of *Choeradoplana.* Accordingly, the diagnosis of the genus is revised below.

##### Choeradoplana eudoxiae

Taxon classificationAnimaliaTricladidaGeoplanidae

Silva & Carbayo sp. nov.

85477301-F2CA-555D-85D2-8A9FD106F588

http://zoobank.org/51DD356F-96DA-43B7-B860-CE6C40B5405B

**Figure 10.**
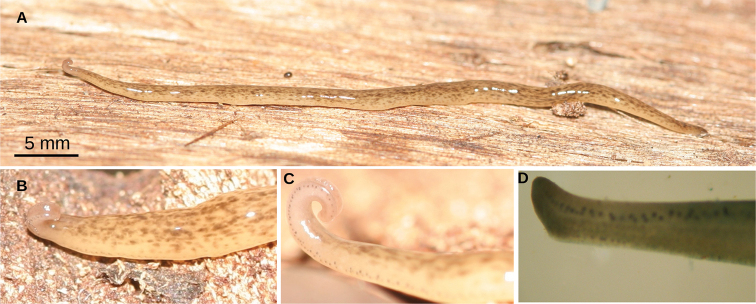
*Choeradoplana eudoxiae* Silva & Carbayo, sp. nov. Holotype **A** living animal in dorsal view **B, C** detail of anterior extremity in a dorsal view **D** detail of anterior extremity in ventro-lateral view in the preserved specimen. Abbreviations: **cm** common muscle coat, **co** common glandular ovovitelline duct, **dd** decussate dorsal cutaneous muscles, **dm** diagonal decussate muscles, **e** eye, **ej** ejaculatory duct, **ep** esophagus, **er** erythrophil secretion, **fa** female atrium, **fd** female genital duct, **g** gonopore, **i** intestine, **lc** longitudinal cutaneous muscles, **ma** male genital atrium, **mk** Muskelgeflecht (Graff, 1899), **mo** mouth, **o** ovary, **ov** ovovitelline duct, **ph** pharyngeal pouch, **pp** penis papilla, **pv** prostatic vesicle, **px** pharynx, **rg** rhabditogen glands, **r** retractor muscle, **sb** subintestinal transverse muscles, **sd** sperm duct, **sg** shell glands, **sk** sunken longitudinal cutaneous muscles, **sm** spermatophore, **sn** subneural transverse muscles, **sp** supra-intestinal transverse muscles, **t** testis, **vi** vitellaria, **vn** ventral nerve plate.

**Figure 11.**
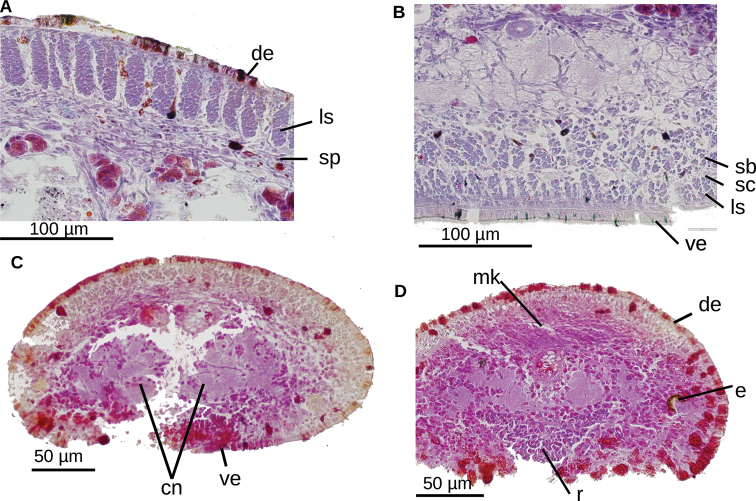
*Choeradoplana eudoxiae* Silva & Carbayo, sp. nov. Photomicrographs of transverse sections of the holotype **A** dorsal portion of the pre-pharyngeal region ofreion **B** ventral portion of the pre-pharyngeal region **C** anterior region at 0.1 mm from the anterior tip of the body. of the pre-pharyngeal region **D** anterior region at 0.5 mm from the anterior tip of the body. Abbreviations: **cm** common muscle coat, **co** common glandular ovovitelline duct, **dd** decussate dorsal cutaneous muscles, **dm** diagonal decussate muscles, **e** eye, **ej** ejaculatory duct, **ep** esophagus, **er** erythrophil secretion, **fa** female atrium, **fd** female genital duct, **g** gonopore, **i** intestine, **lc** longitudinal cutaneous muscles, **ma** male genital atrium, **mk** Muskelgeflecht (Graff, 1899), **mo** mouth, **o** ovary, **ov** ovovitelline duct, **ph** pharyngeal pouch, **pp** penis papilla, **pv** prostatic vesicle, **px** pharynx, **rg** rhabditogen glands, **r** retractor muscle, **sb** subintestinal transverse muscles, **sd** sperm duct, **sg** shell glands, **sk** sunken longitudinal cutaneous muscles, **sm** spermatophore, **sn** subneural transverse muscles, **sp** supra-intestinal transverse muscles, **t** testis, **vi** vitellaria, **vn** ventral nerve plate.

**Figure 12.**
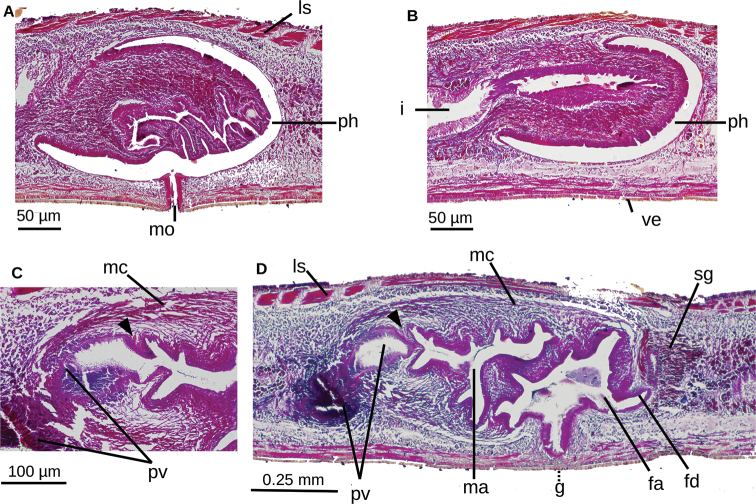
*Choeradoplana eudoxiae* Silva & Carbayo, sp. nov. Photomicrographs of sagittal sections of holotype **A, B** pharynx **C** prostatic vesicle and male atrium **D** copulatory apparatus. Abbreviations: **cm** common muscle coat, **co** common glandular ovovitelline duct, **dd** decussate dorsal cutaneous muscles, **dm** diagonal decussate muscles, **e** eye, **ej** ejaculatory duct, **ep** esophagus, **er** erythrophil secretion, **fa** female atrium, **fd** female genital duct, **g** gonopore, **i** intestine, **lc** longitudinal cutaneous muscles, **ma** male genital atrium, **mk** Muskelgeflecht (Graff, 1899), **mo** mouth, **o** ovary, **ov** ovovitelline duct, **ph** pharyngeal pouch, **pp** penis papilla, **pv** prostatic vesicle, **px** pharynx, **rg** rhabditogen glands, **r** retractor muscle, **sb** subintestinal transverse muscles, **sd** sperm duct, **sg** shell glands, **sk** sunken longitudinal cutaneous muscles, **sm** spermatophore, **sn** subneural transverse muscles, **sp** supra-intestinal transverse muscles, **t** testis, **vi** vitellaria, **vn** ventral nerve plate.

**Figure 13.**
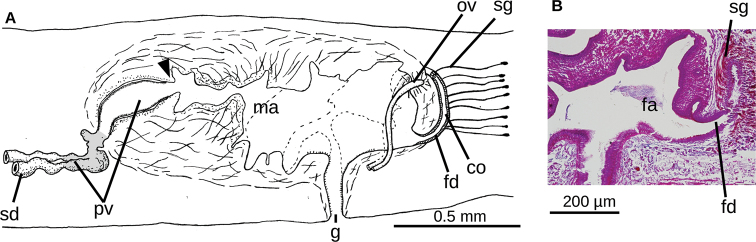
*Choeradoplana eudoxiae* Silva & Carbayo, sp. nov. Holotype **A** diagrammatic reconstruction of copulatory apparatus **B** photomicrograph of a sagittal section of the female atrium. Abbreviations: **cm** common muscle coat, **co** common glandular ovovitelline duct, **dd** decussate dorsal cutaneous muscles, **dm** diagonal decussate muscles, **e** eye, **ej** ejaculatory duct, **ep** esophagus, **er** erythrophil secretion, **fa** female atrium, **fd** female genital duct, **g** gonopore, **i** intestine, **lc** longitudinal cutaneous muscles, **ma** male genital atrium, **mk** Muskelgeflecht (Graff, 1899), **mo** mouth, **o** ovary, **ov** ovovitelline duct, **ph** pharyngeal pouch, **pp** penis papilla, **pv** prostatic vesicle, **px** pharynx, **rg** rhabditogen glands, **r** retractor muscle, **sb** subintestinal transverse muscles, **sd** sperm duct, **sg** shell glands, **sk** sunken longitudinal cutaneous muscles, **sm** spermatophore, **sn** subneural transverse muscles, **sp** supra-intestinal transverse muscles, **t** testis, **vi** vitellaria, **vn** ventral nerve plate.

Figures 10 , 11 , 12 , 13

###### Material examined.

***Holotype***MZUSP 2272 (field code, F3417), sexually mature: Floresta Nacional de São Francisco de Paula, State of Rio Grande do Sul, Brazil, (-29.43628, -50.37369). coll. F. Carbayo and co-workers, 22 January 2009; transverse sections of the cephalic region on 7 slides; horizontal sections of ovaries on 4 slides; transverse sections of the pre-pharyngeal region on 4 slides; sagittal sections of the pharynx and copulatory apparatus on 8 slides; the posterior extremity on 3 slides.

###### Distribution.

Only known from the type locality, Floresta Nacional de São Francisco de Paula, State of Rio Grande do Sul, Brazil.

###### Etymology.

The specific epithet pays homage to the late Prof. Eudóxia Maria Froehlich, 21 October 1928 – 26 September 2015, for her insightful life lessons and lasting contribution to the knowledge of the neotropical planarian fauna for 60 years.

###### Diagnosis.

*Choeradoplana* species with pastel yellow back and brown fawn spots more concentrated in the paramedian region. Its anterior extremity has no lateral dilations or “neck” differentiating its head from its body. The ventral side of the cephalic region is concave, and without distinct glandular cushions. The extrabulbar portion of the prostatic vesicle has paired branches and an unpaired, roughly rounded section; the intrabulbar portion is a dilated vertical duct. Penis papilla is absent.

###### Description.

The live holotype measured 38 mm in length, and 1.5 mm in width. Preserved, it measured 27.5 mm in length and 2 mm in width. Its body is slender and subcylindrical, with the anterior 1/8 becoming progressively thinner towards the anterior tip. The anterior extremity is rounded and the posterior is pointed. The dorsal side is convex, while the ventral side is slightly convex. The anteriormost body portion is approximately five millimeters long and rolled up so that the ventral side is facing upwards (Fig. 10A–C). This ventral surface is concave, without distinct glandular cushions. This ventral surface is flat in the preserved holotype. Its creeping sole is as wide as 75.5% of body width at the pre-pharyngeal region. Its mouth is 14.5 mm (52.7% of body length) from the anterior extremity, and the gonopore is 16.8 mm (61.1%).

The dorsum background color of the body is pastel yellow (RAL 1034) with fawn brown (RAL 8007) spots (Fig. 10A–C); these spots are more densely distributed in the paramedian regions, some merged with each other. The ventral side is cream (Fig. 10D). The cephalic extremity is greyish dorsally and ventrally. The body color faded on the preserved holotype.

The eyes are one pigmented-cup type of 25–30 µm in diameter. There are no clear halos around them (Fig. 10C, D). Since the very anterior histological sections are lost, it could not be ascertained whether they occur in this body region. Posteriorly, the eyes are marginal along the body length.

Sensory pits are 17.0–22.5 µm deep, distributed ventro-laterally in a uniserial row from the anterior sections of the body (approximately 0.2 mm of the anteriormost body were lost) to 4.5 mm behind it.

Abundant rhabditogen cells open onto the dorsal surface of the body and its margins in the pre-pharyngeal region. The epithelium of the margins is also pierced by gland cells producing erythrophil granules (Fig. 11A, B). The ventral epithelium is pierced by three types of gland cells, namely scarce gland cells producing granules of dark, cyanophil secretion, gland cells producing cyanophil granules, and gland cells producing an erythrophil secretion. There is no glandular margin.

The cutaneous musculature consists of a subepithelial circular muscle, followed by a diagonal layer with decussate fibers, and a longitudinal muscle organized in bundles (Fig. 11A, B). This longitudinal muscle is 57.5 μm thick dorsally and arranged in bundles of 50–90 fibers each, whereas ventrally it is divided into a 30 μm-thick muscle of bundles (with 8–15 fibers each), and an insunk muscle with 70 μm-thick bundles (with 16–32 fibers each) (Fig. 11A). The thickness of the cutaneous muscle coat is 20% of the body height.

In the pre-pharyngeal region, the same parenchymal muscles as in *Ch. iheringi*, namely the dorsal decussate muscle (52–55 μm thick), transverse supra-intestinal muscle (20–22 μm); and transverse subintestinal muscle (12–15 μm) (Fig. 11A, B).

The muscle retractor of the head is delta-shaped in a cross-section along ~ 0.5 mm (or 1.8% of body length) starting from 0.1 mm behind the anterior extremity of the body (Fig. 11C, D), and its thickness equals 36% of the height of the cephalic region. The Muskelgeflecht is 32 μm thick (22% of body height). The subneural parenchymal muscle consists of a few transverse fibers. The glandular cushions are composed of a relatively small quantity of rhabditogen cells (Fig. 11C, D).

The central nervous system presents a ventral nerve plate (70–85 μm thick or 9–11% of body height) in the pre-pharyngeal region.

The mouth is located in the middle of the pharyngeal pouch (Fig. 12A). The pharynx is bell-shaped (Fig. 12B). An esophagus is absent. The outer pharyngeal epithelium is underlain by an 8 μm-thick longitudinal muscle, followed by a 15 μm-thick circular muscle. The inner pharyngeal epithelium is underlain by a circular muscle layer with longitudinal fibers interspersed (20 µm thick).

The testes are dorsal, 90–150 μm in diameter, located under the supra-intestinal transverse muscle layer, and partially placed between the intestinal diverticula. The anteriormost testes are located 0.9 mm anterior to the ovaries (or 21% of the body length); posteriormost near the root of the pharynx (49% of the body length). Sperm ducts run immediately above the subintestinal muscle layer, dorsally and slightly internal to the ovovitelline ducts. Distal portions of sperm ducts contain sperm and are surrounded by a 20 μm-thick circular muscle. These ducts communicate with the respective short branch of the prostatic vesicle (Fig. 12C, D). The paired branches run posteriorly. The extrabulbar portion of the prostatic vesicle consists of these paired branches and an unpaired, roughly rounded section with pleated wall receiving the branches. The intrabulbar portion of the prostatic vesicle is elongate and runs dorsally and posteriorly. The passage of the prostatic vesicle to the proximal region of the male atrium is narrowed by an annular fold (Fig. 12C, D). There is no ejaculatory duct, nor penis papilla. The penis bulb is very thick and consists of numerous muscle fibers which are continuous with those underlying the epithelium of the male atrium. The prostatic vesicle is lined with a columnar, ciliated epithelium underlain by a dense layer (20 μm thick) of interwoven circular and longitudinal muscle fibers. The epithelium of the diverticula and that of the anterior section of extrabulbar section of prostatic vesicle are pierced by numerous gland cells producing strong erythrophil (pinkish) granules. The epithelium of the posterior section of the extrabulbar portion is pierced by gland cells producing abundant erythrophil granules. Two types of gland cells pierce the epithelium of the intrabulbar portion of the vesicle; one type is very abundant, and produces cyanophil granules, while the second type produces erythrophil granules.

The male atrium is elongated. The proximal half is horizontal, slightly folded and narrow. The distal half is wider, and provided with two large transverse and oblique folds. The atrium is lined with a 7 μm high epithelium, and pierced by two types of gland cells producing erythrophil and cyanophil granules, respectively. The atrial epithelium is underlain by a 20 to 35 μm-thick circular muscle with longitudinal fibers intermingled (Figs 12D, 13A).

The ovaries are mature, rounded, ~ 100 µm in diameter. They are located 8.9 mm from the anterior extremity of the body (24.7 % of body length), and above the ventral nerve plate. The ovovitelline ducts emerge from the dorso-lateral aspect of the ovaries and run above the nerve plate; their proximal section contains sperm. Laterally to the female atrium, they rise posteriorly to unite dorsally to the common glandular ovovitelline duct (Fig. 13A, B). This common duct is located behind the female atrium, and runs downwards to open into a canalicular projection of the posterior section of the female atrium.

The female atrium is irregular, provided with two or three lateral and dorsal folds (Fig. 13). This atrium is as long as half the male atrium, and is lined with an 8–10 μm high epithelium, the cells of which are erythrophil in their subapical portion. This epithelium is pierced by gland cells producing cyanophil granules, and is underlain by a 10 µm-thick muscle of circular and longitudinal muscle fibers.

The common muscular coat is well developed and continuous with the penis bulb. This coat wraps the intrabulbar portion of the prostatic vesicle, and the male and female atria. The length:height ratio of the copulatory apparatus enveloped by the common muscle coat is 2.2:1.

###### Behavioral note.

When touched with a finger on the posterior end, the animal reacted by rolling forward. Firstly, it lifted its posterior extremity forward to touch the ground at the level of the midbody so that the body forms a loop. Next, the loop moved forwards until the anterior third of the body detached from the ground, which subsequently lengthened and touched the substrate. By doing so, the animal moved forward a distance equivalent to half its body length in approximately one second. It then immediately repeated this whole movement repeatedly.

###### Remarks.

The species described herein matches all diagnostic characteristics of *Choeradoplana*, except for the glandular cushions of the cephalic region, which are not developed. This is so notable that the species was initially assigned to *Cephaloflexa* upon examination of the live and preserved specimen. Regarding the body shape, *Cephaloflexa* is characterized by having “the anterior third very gradually narrowing, without constriction or widening and without grooves on the ventral surface. The anterior tip rolled upwards and is ventrally concave” (Carbayo and Leal-Zanchet 2003). *Ch. albonigra* is similar in this aspect to the genus *Cephaloflexa* and to *Ch. eudoxiae* Silva & Carbayo, sp. nov. Unfortunately, it was not possible to sequence DNA from the small tissue available of *Ch. eudoxiae* Silva & Carbayo, sp. nov., and the homology test of the particular body shape of these two species remains an open question.

The remaining diagnostic attributes of *Choeradoplana* are present in *Ch. eudoxiae* Silva & Carbayo, sp. nov., such as the ventral cutaneous longitudinal muscle partially sunken into the parenchyma; a retractor muscle of the cephalic extremity with a delta shape in transverse section; and a dorsal decussate parenchymatic muscle modified in the cephalic region into the Muskelgeflecht.

The body color of the *Ch. eudoxiae* Silva & Carbayo, sp. nov. resembles that of some congeners in the brownish background color with dark black or dark brown spots over it, namely *Choeradoplana abaiba*Carbayo et al., 2018, *Ch. agua*Carbayo et al., 2018, *Ch. banga* Carbayo & Froehlich, 2012, *Ch. benyai* Lemos & Leal-Zanchet, 2014, *Ch. bocaina* Carbayo & Froehlich, 2012, *Ch. cyanoatria* Iturralde & Leal-Zanchet, 2019, *Ch. longivesicula* Iturralde & Leal-Zanchet, 2019, *Ch. pucupucu*Carbayo et al., 2018, and the herein-described *Ch. onae* Lago-Barcia & Carbayo, sp. nov., *Ch. riutortae* Lago-Barcia & Carbayo, sp. nov. and *Ch. claudioi* Lago-Barcia & Carbayo, sp. nov. However, the general aspect of *Ch. eudoxiae* is lighter. Moreover, only *Ch. eudoxiae* Silva & Carbayo, sp. nov. and *Ch. albonigra* lack the cephalic glandular cushions among all species of the genus, with the latter having a different, black-striped dorsum.

With respect to the internal morphology, *Ch. eudoxiae* Silva & Carbayo, sp. nov. is similar to *Ch. albonigra*, *Ch. tristriata*, *Ch. bocaina*, *Ch. riutortae* Lago-Barcia & Carbayo, sp. nov., *Ch. claudioi* Lago-Barcia & Carbayo, sp. nov., and *Ch. onae* Lago-Barcia & Carbayo, sp. nov. in that the prostatic vesicle is extrabulbar and it is lacking a penis papilla. However, in the latter four species an extrabulbar portion of the prostatic vesicle is dish-shaped, whereas this organ is pear-shaped (in *Ch. eudoxiae* Silva & Carbayo, sp. nov.) or has two short tubular branches (branches (with a pear-shaped organ) *Ch. tristriata* (*paired tubes*) and *Ch. albonigra*). *Ch. eudoxiae* sp. nov. is distinguished from *Ch. tristriata* in the relatively compact copulatory apparatus of the latter, and from *Ch. albonigra* in that the copulatory apparatus in this species is relatively longer, the paired portions of the prostatic vesicle are shorter, and the common glandular ovovitelline duct is relatively shorter.

##### Choeradoplana claudioi

Taxon classificationAnimaliaTricladidaGeoplanidae

Lago-Barcia & Carbayo sp. nov.

CF635688-0922-59C1-B3C7-5C3954F300DE

http://zoobank.org/375D0CE0-B9DF-4AA4-9DEA-1281AFB7BFDC

Figures 14 , 15

###### Material examined.

Both specimens were collected in Reserva Biológica Augusto Ruschi, Santa Teresa, State of Espírito Santo, Brazil (-19.8891, -40.5459) by F. Carbayo and co-workers, May 27–29^th^, 2008; ***Holotype*MZUSP PL 1156** (field code, F2424), sexually mature: transverse sections of the cephalic region on 6 slides; horizontal sections of a portion behind the cephalic region on 4 slides; sagittal sections of the ovarian region on 4 slides; transverse sections of the pre-pharyngeal region on 4 slides; sagittal sections of the pharynx and copulatory apparatus on 11 slides. ***Paratype*MZUSP PL 1157** (field code, F2510), sexually mature: sagittal sections of the pharynx and copulatory apparatus on 10 slides. The ovarian region was lost.

###### Distribution.

Only known from the type locality, Reserva Biológica Augusto Ruschi, Santa Teresa, State of Espírito Santo, Brazil.

###### Etymology.

The specific epithet honors Prof. Claudio Gilberto Froehlich for his contributions to the knowledge of the Neotropical land planarians.

###### Diagnosis.

*Choeradoplana* species with a golden yellow background color, with scattered sepia brown speckles on the whole dorsal surface, except for the anterior, greyish extremity. The extrabulbar portion of the prostatic vesicle is dish-like. The female genital atrium is compressed dorso-ventrally and partially positioned below the distal section of the male atrium.

###### Description.

Preserved specimens measure 24.0–36.5 mm in length and 2.5–3.0 mm in width (n = 2). The body is slender and subcylindrical. The cephalic region is differentiated from the remaining body by a ‘neck’, laterally dilated and rolled up so that the ventral surface provided with glandular cushions faces out; the posterior extremity is pointed. The creeping sole is as wide as 75% (F2424) of the body width at the pre-pharyngeal region. The mouth is positioned at a distance from the anterior extremity equal to 50% of the body length, and the gonopore is at 60%.

**Figure 14.**
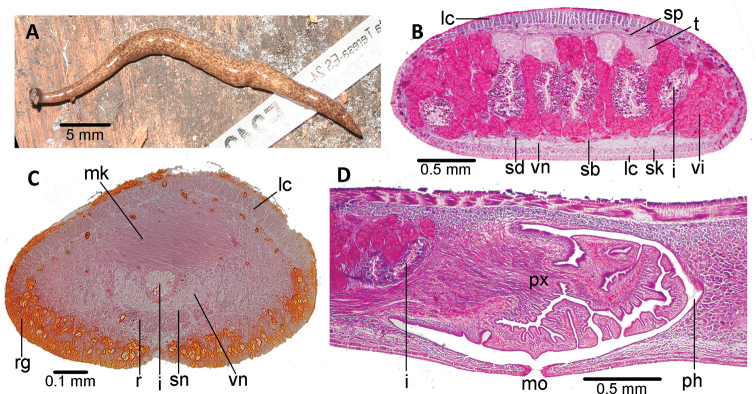
*Choeradoplana claudioi* Lago-Barcia & Carbayo, sp. nov., holotype **A** dorsal view of the creeping live animal **B** photomicrograph of a transverse section of the pre-pharyngeal region **C** photomicrograph of a transverse section of the cephalic region **D** photomicrograph of a sagittal section of the pharynx. Abbreviations: **cm** common muscle coat, **co** common glandular ovovitelline duct, **dd** decussate dorsal cutaneous muscles, **dm** diagonal decussate muscles, **e** eye, **ej** ejaculatory duct, **ep** esophagus, **er** erythrophil secretion, **fa** female atrium, **fd** female genital duct, **g** gonopore, **i** intestine, **lc** longitudinal cutaneous muscles, **ma** male genital atrium, **mk** Muskelgeflecht (Graff, 1899), **mo** mouth, **o** ovary, **ov** ovovitelline duct, **ph** pharyngeal pouch, **pp** penis papilla, **pv** prostatic vesicle, **px** pharynx, **rg** rhabditogen glands, **r** retractor muscle, **sb** subintestinal transverse muscles, **sd** sperm duct, **sg** shell glands, **sk** sunken longitudinal cutaneous muscles, **sm** spermatophore, **sn** subneural transverse muscles, **sp** supra-intestinal transverse muscles, **t** testis, **vi** vitellaria, **vn** ventral nerve plate.

The dorsal coloration of the live specimens consists of a golden yellow (RAL 1004) background color, with scattered sepia brown (RAL 8014) speckles on the whole dorsal surface, except for the anterior, greyish extremity (Fig. 14A). The ventral coloration is golden yellow.

Its eyes are devoid of halos and are formed by a one-pigmented cup of 60 μm in diameter. Eyes are absent in the very anterior extremity of the body equivalent to more or less 1% of the body length. The eyes are distributed marginally in a row of two or three eyes along the first 4.5 mm (or 12% of body length), then they are arranged in a single marginal row until the posterior end.

The sensory pits are 15 µm deep, and are distributed ventro-laterally in a uniserial row, only starting at approximately the equivalent to 1% of body length. The ventral epithelium of the ovarian region was lost and sensory pits are absent in the pre-pharyngeal region.

The cutaneous musculature of the pre-pharyngeal region consists of a subepithelial circular muscle followed by a diagonal layer with decussate fibers, and a strong longitudinal muscle organized in bundles (Fig. 14B). This longitudinal muscle is 95 μm thick dorsally; it is ventrally divided into a 15 μm-thick muscle organized in bundles with 5–12 fibers each, and a 45 μm-thick muscle sunken into the parenchyma constituted of bundles with 6–17 fibers each. The thickness of the cutaneous muscle coat is 16% of the body height. (measurements from animal F2424 which has the best histological sections).

In the pre-pharyngeal region, a dorsal decussate muscle (25 μm thick), transverse supra-intestinal muscle (25 μm); and transverse subintestinal muscle (15 μm) (n = 1) (Fig. 14B).

The cutaneous and parenchymal musculature is organized in the cephalic region as in *Ch. iheringi*. The muscle retractor of the head is delta-shaped in a cross-section along 1.8 mm (or 5% of body length, F2424) from behind, 1.3 mm (or 4%, F2424) of the anterior extremity of the body (Fig. 14C), and its thickness equals 36% of the height of the cephalic region. The Muskelgeflecht is 190 μm thick (32% of body height). The subneural parenchymal muscle consists of scattered transverse fibers. The glandular cushions are composed of numerous rhabditogen cells (Fig. 14C).

The mouth is located in the middle of the pharyngeal pouch (Fig. 14D). The pharynx is cylindrical-to-bell-shaped, with its dorsal insertion approximately at the mouth level. An esophagus is absent. The pharyngeal pouch is lined with a non-ciliated, low epithelium underlain by a thin layer of circular muscle with interspersed longitudinal fibers (11–12 μm thick, n = 2). The outer pharyngeal epithelium is flat, ciliated and underlain by circular muscle (40–48 μm thick, n = 2) with interspersed longitudinal fibers ectally. The inner pharyngeal epithelium is flat, ciliated, and underlain by a thin circular muscle (48–50 μm, n = 2). The pharynx presents numerous xanthophil, erythrophil and cyanophil gland cells.

**Figure 15.**
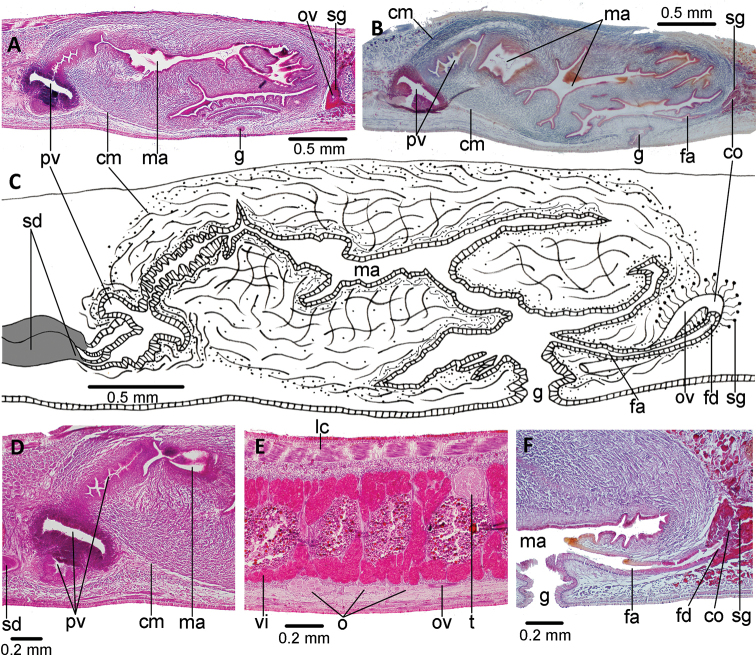
*Choeradoplana claudioi* Lago-Barcia & Carbayo, sp. nov. **A** photomicrograph of a sagittal section of the copulatory apparatus of holotype **B** photomicrograph of a sagittal section of the copulatory apparatus of paratype F2510 **C** diagrammatic representation of the copulatory apparatus of holotype **D** photomicrograph of a sagittal section of the prostatic vesicle of holotype **E** photomicrograph of a sagittal section of the ovarian region of holotype **F** photomicrograph of a sagittal section of the female atrium of holotype. Abbreviations: **cm** common muscle coat, **co** common glandular ovovitelline duct, **dd** decussate dorsal cutaneous muscles, **dm** diagonal decussate muscles, **e** eye, **ej** ejaculatory duct, **ep** esophagus, **er** erythrophil secretion, **fa** female atrium, **fd** female genital duct, **g** gonopore, **i** intestine, **lc** longitudinal cutaneous muscles, **ma** male genital atrium, **mk** Muskelgeflecht (Graff, 1899), **mo** mouth, **o** ovary, **ov** ovovitelline duct, **ph** pharyngeal pouch, **pp** penis papilla, **pv** prostatic vesicle, **px** pharynx, **rg** rhabditogen glands, **r** retractor muscle, **sb** subintestinal transverse muscles, **sd** sperm duct, **sg** shell glands, **sk** sunken longitudinal cutaneous muscles, **sm** spermatophore, **sn** subneural transverse muscles, **sp** supra-intestinal transverse muscles, **t** testis, **vi** vitellaria, **vn** ventral nerve plate.

The testes are mature, dorsal, arranged in four paramedian rows between the supra-intestinal transverse parenchymal muscle and the intestinal diverticula (Fig. 14B). They extend from the level of the ovaries (i.e., 7.7 mm behind the anterior extremity of the body, or 21% of body length, holotype) to the root of the pharynx (48%). Sperm ducts run immediately above the subintestinal parechymatic muscle layer. In their distal portion, they open into the respective branch of the prostatic vesicle (Fig. 15A–C). The prostatic vesicle is divided into two differentiated halves (Fig. 15A–D). The proximal half is extrabulbar and constituted by the two widened and rounded branches opening into a broadened, dish-shaped section located above the paired portion. The distal half is intrabulbar, dilated canal oriented dorso-posteriorly. The paired portion is lined with a cuboidal-to-columnar, ciliated epithelium, which is pierced by numerous gland cells producing fine erythrophil granules. The columnar epithelium of the dish-shaped portion is pierced by very numerous gland cells producing erythrophil gross granules (1–2 μm); and by two types of scarce gland cells producing fine, erythrophil and xanthophil granules, respectively (Fig. 15D). The distal half is lined by a columnar, ciliated epithelium with a sinuous surface which is pierced by gland cells producing erythrophil granules along its whole length, and additionally a low number of gland cells producing xanthophil granules in its distal portion. The lining epithelium of the proximal half of the prostatic vesicle is coated by a 28–30 µm-thick (n = 2) circular muscle; the distal half is coated by a 1 µm-thick circular muscle, followed by a 22–25 µm-thick (n = 2) longitudinal muscle. The extrabulbar portion of the prostatic vesicle is coated by additional muscle fibers attaching it to the common muscle coat (Fig. 15A–C). The opening of the prostatic vesicle into the antero-dorsal region of the male atrium is wide, without an ejaculatory duct or penis papilla (Fig. 15A–C).

The male atrium is 5–6× longer than the female atrium, and divided into a dorsal, proximal narrow third, slightly folded, and a distal two-thirds portion with some smaller folds. A main, very large, oblique fold on each side of the body extends behind the gonopore level and over the female atrium (Fig. 15E). The male atrium is lined by a cuboidal, non-ciliated epithelium, and is underlain by a 30–60 µm-thick mixed layer of circular muscle with numerous interspersed longitudinal fibers (n = 2). The whole atrium receives two types of abundant gland cells producing erythrophil and cyanophil fine granules, respectively, and a third type of gland cells producing amorphous xanthophil secretion in the proximal third of the atrium.

The ovaries are mature, very elongated and placed above the ventral nerve plate at a distance from anterior tip of the body equal to 21% of body length (7.7 mm from anterior tip) (holotype). They present an anterior, ovoid section, 300 μm in length (F2424), and a posterior, 600 µm (F2424) long narrow section (Fig. 15E). Ovovitelline ducts emerge from the lateral aspect of the ovoid section of the ovaries and run ventrally. Lateral to the posterior section of the female atrium, the ovovitelline ducts run medially and dorsally, then unite posteriorly to the female atrium (Fig. 15C). The common glandular ovovitelline duct is 45–50 μm in length (n = 2) and runs ventro-anteriorly to communicate with the female genital canal. This canal runs slightly downwards and anteriorly, subsequently penetrates the common muscle coat to open into the female atrium. The female genital canal is lined by a cuboidal, ciliated epithelium.

The female atrium is dorso-ventrally compressed and wider towards the gonopore canal. It is placed below the posterior section of the male atrium (Fig. 15F), and is lined with a cuboidal non-ciliated epithelium. This epithelium is pierced by gland cells producing fine xanthophil granules. The lining epithelium of the female atrium is underlain by a 37 μm-thick layer of mixed circular and longitudinal muscle fibers (n = 2).

The common muscle coat is a very dense layer composed by variously oriented muscle fibers. The length:height ratio of the copulatory apparatus enveloped by the common muscle coat ranges between 2.5–2.8:1.

###### Remarks.

*Ch. claudioi* Lago-Barcia & Carbayo, sp. nov. externally differs from most congeners in that the dorsum is composed of a light background color evenly covered with brown spots. However, this color pattern is so similar to *Ch. abaiba*, *Ch. agua*, *Ch. banga*, *Ch. iheringi*, and *Ch. pucupucu* that *Ch. claudioi* Lago-Barcia & Carbayo, sp. nov. cannot be confidently distinguished from them.

With respect to the internal morphology, *Ch. claudioi* Lago-Barcia & Carbayo, sp. nov. can be differentiated from most *Choeradoplana* species by the dish-shaped portion of the extrabulbar region of the prostatic vesicle. This attribute is only shared with *Ch. onae* Lago-Barcia & Carbayo, sp. nov., *Ch. riutortae* Lago-Barcia & Carbayo, sp. nov., and *Ch. bocaina*. However, the female genital atrium is compressed dorso-ventrally and partially positioned below the distal section of the male atrium, which readily distinguishes *Ch. claudioi* Lago-Barcia & Carbayo, sp. nov. from these three other species.

##### Choeradoplana onae

Taxon classificationAnimaliaTricladidaGeoplanidae

Lago-Barcia & Carbayo sp. nov.

B1793531-B93C-5564-9588-8B229AD87CC5

http://zoobank.org/0CBCD7FD-683B-4075-9D90-B3C62CB97631

**Figure 16.**
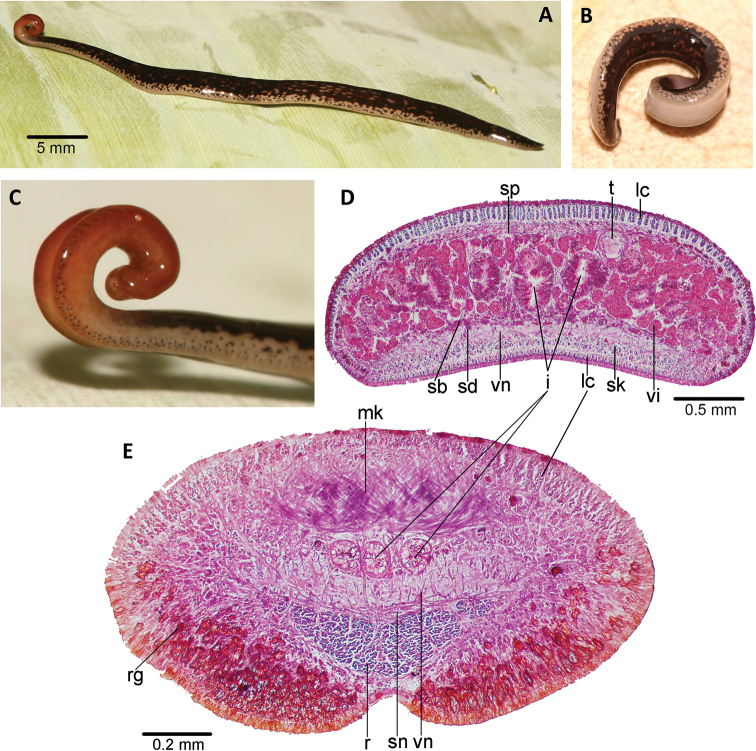
*Choeradoplana onae* Lago-Barcia & Carbayo, sp. nov. **A** dorsal view of the creeping live paratype F2230 **B** live paratype F2310 rolled up showing the ventral surface **C** lateral view of the cephalic region of live paratype F2230 **D** photomicrograph of a transverse section of the pre-pharyngeal region of holotype **E** photomicrograph of a transverse section of the cephalic region of holotype. Abbreviations: **cm** common muscle coat, **co** common glandular ovovitelline duct, **dd** decussate dorsal cutaneous muscles, **dm** diagonal decussate muscles, **e** eye, **ej** ejaculatory duct, **ep** esophagus, **er** erythrophil secretion, **fa** female atrium, **fd** female genital duct, **g** gonopore, **i** intestine, **lc** longitudinal cutaneous muscles, **ma** male genital atrium, **mk** Muskelgeflecht (Graff, 1899), **mo** mouth, **o** ovary, **ov** ovovitelline duct, **ph** pharyngeal pouch, **pp** penis papilla, **pv** prostatic vesicle, **px** pharynx, **rg** rhabditogen glands, **r** retractor muscle, **sb** subintestinal transverse muscles, **sd** sperm duct, **sg** shell glands, **sk** sunken longitudinal cutaneous muscles, **sm** spermatophore, **sn** subneural transverse muscles, **sp** supra-intestinal transverse muscles, **t** testis, **vi** vitellaria, **vn** ventral nerve plate.

**Figure 17.**
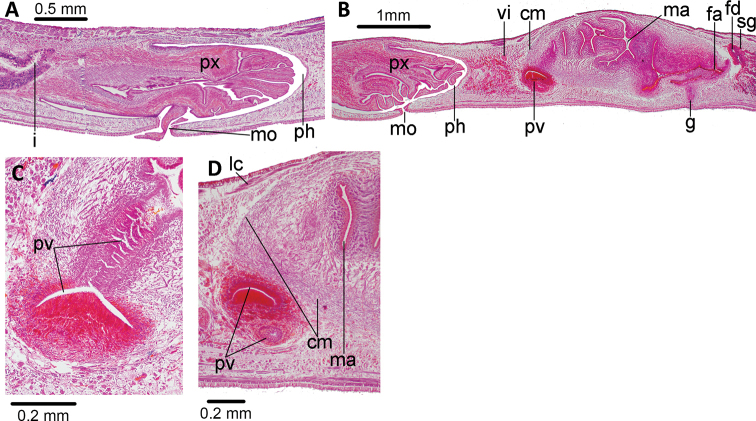
*Choeradoplana onae* Lago-Barcia & Carbayo, sp. nov. Photomicrographs of sagittal sections **A** pharynx of paratype F2230 **B** pharynx and copulatory apparatus of holotype **C** prostatic vesicle of paratype F2230 **D** prostatic vesicle of holotype. Abbreviations: **cm** common muscle coat, **co** common glandular ovovitelline duct, **dd** decussate dorsal cutaneous muscles, **dm** diagonal decussate muscles, **e** eye, **ej** ejaculatory duct, **ep** esophagus, **er** erythrophil secretion, **fa** female atrium, **fd** female genital duct, **g** gonopore, **i** intestine, **lc** longitudinal cutaneous muscles, **ma** male genital atrium, **mk** Muskelgeflecht (Graff, 1899), **mo** mouth, **o** ovary, **ov** ovovitelline duct, **ph** pharyngeal pouch, **pp** penis papilla, **pv** prostatic vesicle, **px** pharynx, **rg** rhabditogen glands, **r** retractor muscle, **sb** subintestinal transverse muscles, **sd** sperm duct, **sg** shell glands, **sk** sunken longitudinal cutaneous muscles, **sm** spermatophore, **sn** subneural transverse muscles, **sp** supra-intestinal transverse muscles, **t** testis, **vi** vitellaria, **vn** ventral nerve plate.

**Figure 18.**
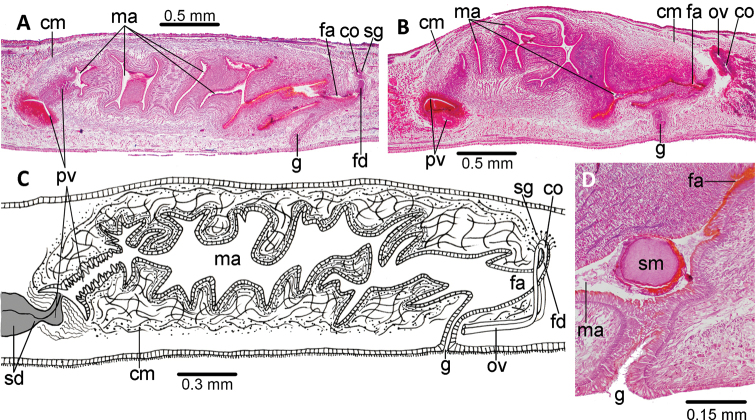
*Choeradoplana onae* Lago-Barcia & Carbayo, sp. nov. **A** photomicrograph of a sagittal section of the copulatory apparatus of paratype F2230 **B** photomicrograph of a sagittal section of the copulatory apparatus of holotype **C** diagrammatic representation of the copulatory apparatus of paratype F2230 **D** photomicrograph of a sagittal section of the entrance of the gonopore of paratype F2281 housing a spermatophore. Abbreviations: **cm** common muscle coat, **co** common glandular ovovitelline duct, **dd** decussate dorsal cutaneous muscles, **dm** diagonal decussate muscles, **e** eye, **ej** ejaculatory duct, **ep** esophagus, **er** erythrophil secretion, **fa** female atrium, **fd** female genital duct, **g** gonopore, **i** intestine, **lc** longitudinal cutaneous muscles, **ma** male genital atrium, **mk** Muskelgeflecht (Graff, 1899), **mo** mouth, **o** ovary, **ov** ovovitelline duct, **ph** pharyngeal pouch, **pp** penis papilla, **pv** prostatic vesicle, **px** pharynx, **rg** rhabditogen glands, **r** retractor muscle, **sb** subintestinal transverse muscles, **sd** sperm duct, **sg** shell glands, **sk** sunken longitudinal cutaneous muscles, **sm** spermatophore, **sn** subneural transverse muscles, **sp** supra-intestinal transverse muscles, **t** testis, **vi** vitellaria, **vn** ventral nerve plate.

Figures 16 , 17 , 18

Choeradoplana sp. in Álvarez-Presas et al. 2014.

###### Material examined.

All specimens collected in the Reserva Biológica Augusto Ruschi, Santa Teresa, State of Espírito Santo, Brazil (-19.88, -40.54) by F. Carbayo and co-workers, 25–27 May 2008; ***Holotype*MZUSP PL 2270** (field code, F2414), transverse sections of the cephalic region on 8 slides; horizontal sections of the portion behind the cephalic region on 5 slides; sagittal sections of the ovarian region on 12 slides; horizontal sections of the testes on 10 slides; transverse sections of the pre-pharyngeal region on 9 slides; sagittal sections of the pharynx and copulatory apparatus on 12 slides; ***Paratype*MZUSP PL 2267** (field code, F2230), transverse sections of the cephalic region on 8 slides; sagittal sections of the pharynx and copulatory apparatus on 9 slides; ***Paratype*MZUSP PL 2268** (field code, F2281), sagittal sections of the pharynx and copulatory apparatus on 12 slides. ***Paratype*MZUSP PL** 2269 (field code, F2310), transverse sections of the cephalic region on 12 slides; horizontal sections of the ovarian region on 8 slides.

###### Distribution.

Only known from the type locality, Reserva Biológica Augusto Ruschi, Santa Teresa, State of Espírito Santo, Brazil.

###### Etymology.

The name *onae* is the affectionate nickname of the biologist Marta Álvarez-Presas (Bristol University). The specific epithet honors her for her contributions to understanding the systematics of free-living flatworms.

###### Diagnosis.

*Choeradoplana* species with a light ivory background color and a wide sepia brown median band. The extrabulbar region of the prostatic vesicle has a dish-shaped portion. The copulatory apparatus is 3.8× longer than its height. The male atrium has 4–6 main folds.

###### Description.

**External aspect.** Preserved specimens range between 41–44.5 mm in length and 3–4 mm (n = 4) in width. The body is slender and subcylindrical. The cephalic region is differentiated from the remaining body by a ‘neck’ and laterally dilated. This region is rolled up so that the ventral surface provided with two prominent glandular cushions is facing out when alive (Fig. 16A–C); the posterior extremity is pointed. The creeping sole is as wide as 85–87% of body width at the pre-pharyngeal region (n = 4). The mouth is positioned at a distance from the anterior extremity equal to 51% of body length, and the gonopore is 61% (paratype F2230).

The dorsal coloration of live specimens consists of a light ivory (RAL 1015) background color (Fig. 16A), covered on a wide median band with sepia brown pigment (RAL 8014), except for irregular clear spots with the background color exposed. The bordering line of the band merging with the background color on the sides is irregular with large sepia brown spots. The curled anterior extremity is red orange (RAL 2001). The ventral surface is red orange in the cephalic region, and light grey (RAL 7035) in the rest of body (Fig. 16B).

The eyes are devoid of halos, and formed by a one-pigmented cup of 60 μm in diameter. Eyes are absent in the very anterior extremity of the body equivalent to 1% of the body length (n = 1). Eyes behind the anterior tip are distributed marginally in a row of two or three eyes (Fig. 5C) and extend along the entire body until the posterior end.

Sensory pits are 20–25 µm deep in a uniserial ventro-lateral row, starting from 0.4 mm behind the anterior extremity, the equivalent of 1% body length to at least 80 mm from the anterior tip (20% of body length, n = 1).

Rhabditogen gland cells pierce the marginal epidermis in the pre-pharyngeal region. Erythrophil granules and scarce cyanophil granules are discharged through the entire epidermis. There is no glandular margin (Fig. 16D).

The cutaneous musculature consists of a subepithelial circular muscle, followed by a diagonal layer with decussate fibers, and a strong longitudinal muscle organized in bundles (Fig. 16D). This longitudinal muscle is 90–100 μm thick dorsally, and organized in tight bundles with > 32–60 fibers each; it is ventrally divided into a 30–32.5 μm-thick muscle organized in bundles with 5–11 fibers each, and a 75 μm-thick muscle sunken into the parenchyma, and constituted of bundles with 8–27 fibers each (Fig. 16D). The thickness of the cutaneous muscle coat is 18–20% (n = 4) of the body height.

In the pre-pharyngeal region, there are three parenchymal muscles, namely a dorsal decussate muscle (40–50 μm thick), a transverse supra-intestinal muscle (15 μm); and transverse subintestinal muscle (18–20 μm) (n = 4) (Fig. 16D).

The cutaneous and parenchymal musculature is organized in the cephalic region as in *Ch. iheringi*. The muscle retractor of the head is delta-shaped in a cross-section along 2.5 mm (or 6% of body length) from behind, 0.9 mm (or 2%) anterior extremity of the body (Fig. 16E), and its thickness equals 19% of the height of the cephalic region. The Muskelgeflecht is 160–180 μm thick (23% of body height). The subneural parenchymal muscle consists of a number of transverse fibers; this muscle is weak in the ovarian region. Glandular cushions are composed of numerous rhabditogen cells (Fig. 16E).

The mouth is located in the middle of the pharyngeal pouch (n = 4) (Fig. 17A, B). The pharynx is bell-shaped with its dorsal insertion at mouth level (n = 4). The esophagus is as long as 15% of the pharyngeal length. The pharyngeal pouch is lined with a non-ciliated, low epithelium underlain by a one-fiber thick longitudinal muscle followed by a 10 µm-thick circular muscle. The outer pharyngeal epithelium is flat, ciliated, and underlain by a 5 µm-thick longitudinal muscle followed by a 15 µm-thick circular muscle. The inner pharyngeal epithelium is flat and ciliated, and is underlain by a mixed circular muscle with longitudinal fibers (80 μm thick). The pharynx has numerous interspersed erythrophil and xanthophil gland cells.

The testes are mature, dorsally located under the supra-intestinal transverse parenchymal muscle, placed between the intestinal diverticula. They extend from 13.2 mm (32% of body length, holotype) from the anterior extremity to 0.2 mm of the root of the pharynx (63%, holotype). Sperm ducts bend dorsally and medially immediately above the subintestinal parechymatic muscle layer to open into the respective dilated branch of the prostatic vesicle. The prostatic vesicle is divided into two halves (Fig. 17C). The anterior half is extrabulbar and proximally presents a dilated and paired tubular portion oriented vertically which opens into a broadened, dish-shaped section located above the paired portion. This proximal half is lined by a columnar-to-cuboidal epithelium that is pierced by gland cells producing xanthophil granules. These gland cells are much more abundant in the dish-shaped portion and present a strongly reddish appearance; the ventral face and the border of this dish-shaped section of the prostatic vesicle is also pierced by gland cells producing cyanophil granules. The distal half is an intrabulbar dilated canal oriented dorso-posteriorly. It is lined by a columnar epithelium with a sinuous surface that is pierced by gland cells producing cyanophil granules along its whole length and additionally gland cells producing xanthophil granules in its distal portion. The lining epithelium of the proximal half of prostatic vesicle is coated by an 18–20 µm-thick circular muscle layer, while it is coated by a 1 µm-thick circular muscle in the distal half, followed by a 15 µm-thick longitudinal muscle (n = 4). The extrabulbar portion of the prostatic vesicle is coated by additional muscle fibers attaching it to the common muscle coat (Fig. 17D). The opening of the prostatic vesicle into the antero-dorsal region of the male atrium is wide, without an ejaculatory duct or penis papilla (Fig. 18A–C).

The male atrium is long, 5× as long as the female atrium, with the same height along its length and 4–6 large transverse folds narrowing its lumen. The male atrium is lined by a cuboidal, non-ciliated epithelium, and is underlain by a 40–70 µm-thick circular muscle with numerous interspersed longitudinal fibers (n = 4). The proximal two thirds of the atrium receive two types of abundant gland cells producing xanthophil and erythrophil granules, respectively, and a third type of scarce gland cells producing amorphous xanthophil secretion; the distal third of the male atrium receives abundant gland cells producing erythrophil granules. The sub-apical portion of the cells of the lining epithelium of this distal third contains xanthophil granules.

The ovaries are mature, ovoid, 250 μm in length, placed above the ventral nerve plate and at a distance from the anterior tip of the body equal to 28% of body length (11.8 mm from the anterior tip) (n = 1). Ovovitelline ducts emerge from the dorso-lateral aspect of the ovaries and run ventrally. Ovovitelline ducts run medially and dorsally lateral to the posterior section of the female atrium, then unite above the postero-dorsal section of the female atrium (Fig. 18A–C). The common glandular ovovitelline duct length ranges between 25–150 µm in length (n = 4) and runs ventrally or ventro-posteriorly to communicate with the female genital canal. This canal runs downwards, and subsequently penetrates the common muscle coat to open into the posterior section of the female atrium. The genital canal is lined by a cuboidal, ciliated epithelium, and the sub-apical portion of its lining cells is stained reddish.

The female atrium is funnel-shaped, and is lined with a 50 µm high epithelium, which is pierced by gland cells producing fine erythrophil granules. The subapical portion of the lining cells contains xanthophil granules. The lining epithelium of the female atrium is underlain by a 1-fiber-thick longitudinal muscle, followed by a 10 µm-thick layer of decussate muscle fibers. Paratype F2281 presents a female atrium smaller than that of the remaining specimens and also bears a spermatophore at the entrance of the gonopore canal. This spermatophore is ovoid, and with approximately 100 µm in maximum diameter. It is constituted on an inner mass of sperm surrounded by a thin fibrous, erythrophil layer, external to which is a gross layer of xanthophil, granular secretion and a bluish fine granular secretion, each prevailing on one side of the spermatophore (Fig. 18D).

The common muscle coat is a very dense layer composed by variously oriented muscle fibers. The length:height ratio of the copulatory apparatus enveloped by the common muscle coat ranges between 2.5–3.3:1 (n = 3).

###### Remarks.

The species described herein matches all diagnostic characteristics of *Choeradoplana*. As reported for *Ch. claudioi* Lago-Barcia & Carbayo, sp. nov., *Ch. onae* Lago-Barcia & Carbayo, sp. nov. only resembles *Ch. abaiba*, *Ch. agua*, *Ch. banga*, *Ch. iheringi*, *Ch. claudioi* Lago-Barcia & Carbayo, sp. nov., and *Ch. pucupucu* in the body color. The great similarity between them hinders confident identification. However, none of them present the prominent cushions found in this species with a red-orange color.

With respect to the internal morphology, *Ch. onae* Lago-Barcia & Carbayo, sp. nov. only compares with *Ch. riutortae* Lago-Barcia & Carbayo, sp. nov. and *Ch. bocaina* in that they also present a dish-shaped prostatic vesicle. However, the length:height ratio of the copulatory apparatus in these species is 2.6:1 (vs. 3.8:1 in *Ch. onae* Lago-Barcia & Carbayo, sp. nov.); the male atrium:female atrium ratio in *Ch. bocaina* and *Ch. riutortae* Lago-Barcia & Carbayo, sp. nov. ratio ranges between 1:1–3:1 (Carbayo & E. M. Froehlich, 2012), whereas it is ~5:1 in *Ch. claudioi* Lago-Barcia & Carbayo, sp. nov. and *Ch. onae* Lago-Barcia & Carbayo, sp. nov., and the male atrium presents 1–2 large folds (vs. 4–6 in *Ch. onae* Lago-Barcia & Carbayo, sp. nov.).

##### Choeradoplana riutortae

Taxon classificationAnimaliaTricladidaGeoplanidae

Lago-Barcia & Carbayo sp. nov.

417B6F4F-6FE1-57D0-BF59-4B5A03647F83

http://zoobank.org/DDF3C826-CC45-4D40-8438-602D8BC687CF

**Figure 19.**
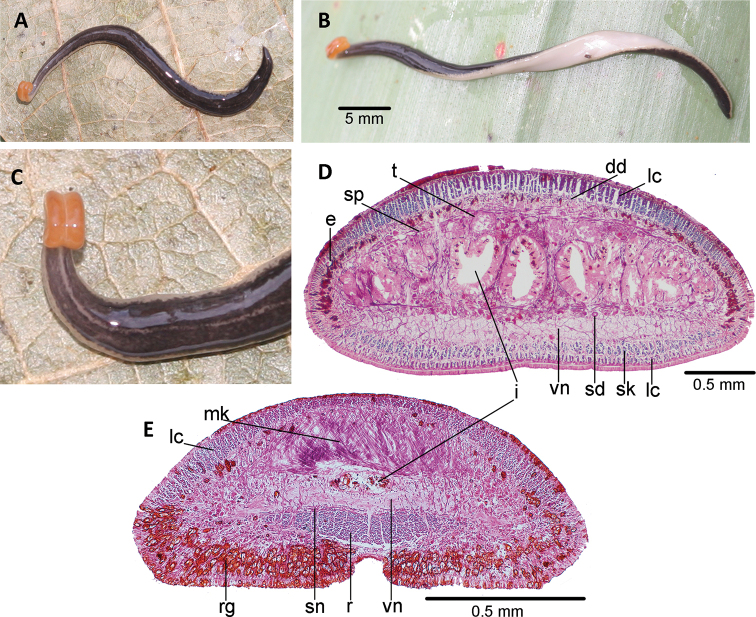
*Choeradoplana riutortae* Lago-Barcia & Carbayo, sp. nov. **A** dorsal view of the creeping live paratype F4217 **B** the live holotype twisted showing the ventral surface **C** dorsal view of the anterior region of paratype F4217 **D** photomicrograph of a transverse section of the pre-pharyngeal region of holotype **E** photomicrograph of a transverse section of the cephalic region of holotype. Abbreviations: **cm** common muscle coat, **co** common glandular ovovitelline duct, **dd** decussate dorsal cutaneous muscles, **dm** diagonal decussate muscles, **e** eye, **ej** ejaculatory duct, **ep** esophagus, **er** erythrophil secretion, **fa** female atrium, **fd** female genital duct, **g** gonopore, **i** intestine, **lc** longitudinal cutaneous muscles, **ma** male genital atrium, **mk** Muskelgeflecht (Graff, 1899), **mo** mouth, **o** ovary, **ov** ovovitelline duct, **ph** pharyngeal pouch, **pp** penis papilla, **pv** prostatic vesicle, **px** pharynx, **rg** rhabditogen glands, **r** retractor muscle, **sb** subintestinal transverse muscles, **sd** sperm duct, **sg** shell glands, **sk** sunken longitudinal cutaneous muscles, **sm** spermatophore, **sn** subneural transverse muscles, **sp** supra-intestinal transverse muscles, **t** testis, **vi** vitellaria, **vn** ventral nerve plate.

**Figure 20.**
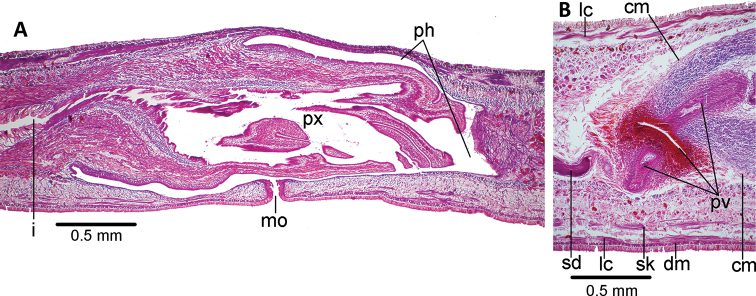
*Choeradoplana riutortae* Lago-Barcia & Carbayo, sp. nov., holotype. Photomicrographs of sagittal sections **A** pharynx **B** prostatic vesicle. Abbreviations: **cm** common muscle coat, **co** common glandular ovovitelline duct, **dd** decussate dorsal cutaneous muscles, **dm** diagonal decussate muscles, **e** eye, **ej** ejaculatory duct, **ep** esophagus, **er** erythrophil secretion, **fa** female atrium, **fd** female genital duct, **g** gonopore, **i** intestine, **lc** longitudinal cutaneous muscles, **ma** male genital atrium, **mk** Muskelgeflecht (Graff, 1899), **mo** mouth, **o** ovary, **ov** ovovitelline duct, **ph** pharyngeal pouch, **pp** penis papilla, **pv** prostatic vesicle, **px** pharynx, **rg** rhabditogen glands, **r** retractor muscle, **sb** subintestinal transverse muscles, **sd** sperm duct, **sg** shell glands, **sk** sunken longitudinal cutaneous muscles, **sm** spermatophore, **sn** subneural transverse muscles, **sp** supra-intestinal transverse muscles, **t** testis, **vi** vitellaria, **vn** ventral nerve plate.

**Figure 21.**
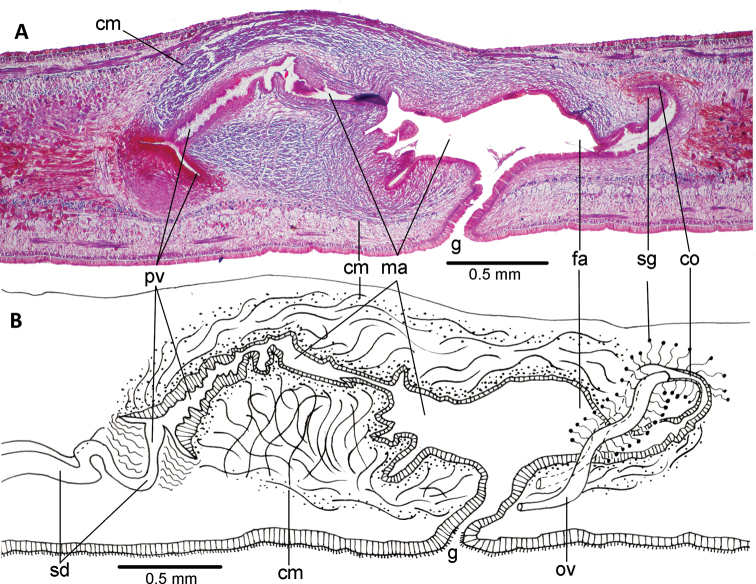
*Choeradoplana riutortae* Lago-Barcia & Carbayo, sp. nov., holotype **A** photomicrograph of a sagittal section of the copulatory apparatus **C** Diagrammatic representation of the copulatory apparatus. Abbreviations: **cm** common muscle coat, **co** common glandular ovovitelline duct, **dd** decussate dorsal cutaneous muscles, **dm** diagonal decussate muscles, **e** eye, **ej** ejaculatory duct, **ep** esophagus, **er** erythrophil secretion, **fa** female atrium, **fd** female genital duct, **g** gonopore, **i** intestine, **lc** longitudinal cutaneous muscles, **ma** male genital atrium, **mk** Muskelgeflecht (Graff, 1899), **mo** mouth, **o** ovary, **ov** ovovitelline duct, **ph** pharyngeal pouch, **pp** penis papilla, **pv** prostatic vesicle, **px** pharynx, **rg** rhabditogen glands, **r** retractor muscle, **sb** subintestinal transverse muscles, **sd** sperm duct, **sg** shell glands, **sk** sunken longitudinal cutaneous muscles, **sm** spermatophore, **sn** subneural transverse muscles, **sp** supra-intestinal transverse muscles, **t** testis, **vi** vitellaria, **vn** ventral nerve plate.

**Figure 22.**
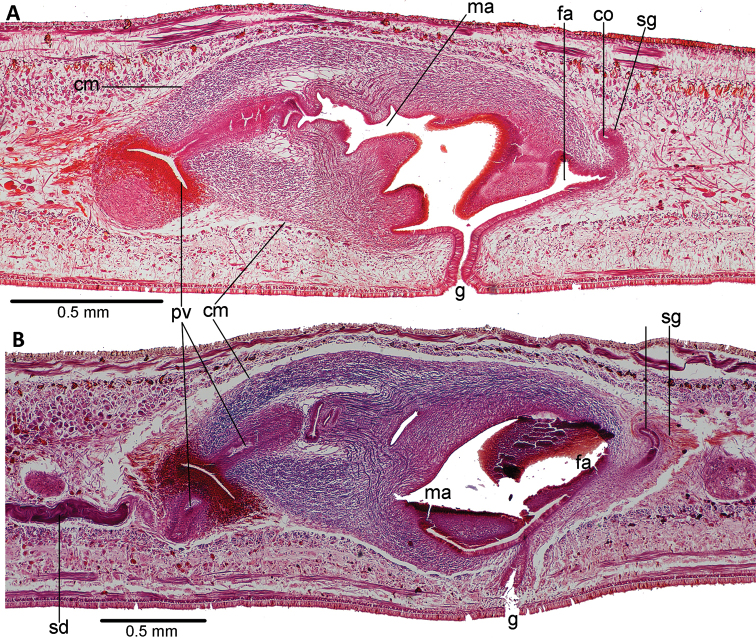
*Choeradoplana riutortae* Lago-Barcia & Carbayo, sp. nov. Photomicrographs of sagittal sections **A** copulatory apparatus of paratype F4217 **B** copulatory apparatus of paratype F4261. Abbreviations: **cm** common muscle coat, **co** common glandular ovovitelline duct, **dd** decussate dorsal cutaneous muscles, **dm** diagonal decussate muscles, **e** eye, **ej** ejaculatory duct, **ep** esophagus, **er** erythrophil secretion, **fa** female atrium, **fd** female genital duct, **g** gonopore, **i** intestine, **lc** longitudinal cutaneous muscles, **ma** male genital atrium, **mk** Muskelgeflecht (Graff, 1899), **mo** mouth, **o** ovary, **ov** ovovitelline duct, **ph** pharyngeal pouch, **pp** penis papilla, **pv** prostatic vesicle, **px** pharynx, **rg** rhabditogen glands, **r** retractor muscle, **sb** subintestinal transverse muscles, **sd** sperm duct, **sg** shell glands, **sk** sunken longitudinal cutaneous muscles, **sm** spermatophore, **sn** subneural transverse muscles, **sp** supra-intestinal transverse muscles, **t** testis, **vi** vitellaria, **vn** ventral nerve plate.

Figures 19 , 20 , 21 , 22

###### Material examined.

All specimens were collected in the Parque Nacional da Serra dos Orgãos, Teresópolis, State of Rio de Janeiro, Brazil (-22.48, -43.06) by F. Carbayo and co-workers, January 6^th^, 2010. ***Holotype*MZUSP 2274** (field code, F4218), transverse sections of the cephalic region on 5 slides; horizontal sections of the portion behind the cephalic region on 6 slides; sagittal sections of ovarian region on 6 slides; horizontal sections of the testes on 4 slides; transverse sections of the pre-pharyngeal region on 7 slides; sagittal sections of the pharynx and copulatory apparatus on 9 slides. ***Paratype*MZUSP PL 1174** (field code, F4217), transverse sections of the cephalic region on 9 slides; horizontal sections of the ovarian region on 7 slides; transverse sections of the pre-pharyngeal region on 8 slides; sagittal sections of the pharynx and copulatory apparatus on 12 slides. ***Paratype*MZUSP PL 2275** (field code, F4261), transverse sections of the pre-pharyngeal region on 16 slides; sagittal sections of the pharynx and copulatory apparatus on 27 slides.

###### Distribution.

Only known from the type locality, Parque Nacional da Serra dos Orgãos, municipality of Teresópolis, State of Rio de Janeiro, Brazil.

###### Etymology.

The specific epithet honors Prof. Marta Riutort for her contributions to understanding the evolution of flatworms.

###### Diagnosis.

*Choeradoplana* species with a light ivory background color covered by numerous sepia brown spots except for the anterior extremity which is red orange. The ventral surface is pale orange in the cephalic region, and light grey in the rest of the body. Part of the longitudinal cutaneous musculature is sunken in the parenchyma of the ventral side. The prostatic vesicle has a paired extrabulbar dish-shaped portion, and an elongated intrabulbar portion with an irregular epithelium. It has a short copulatory apparatus (the length:height ratio of the copulatory apparatus is 2.6:1). The male atrium presents the same size as the female atrium.

###### Description.

The preserved animals measure between 37–42 mm in length and 2.5–3 mm in width (n = 3). The body is slender and subcylindrical. The cephalic region is differentiated from the remaining body by a ‘neck’, laterally dilated and rolled up so that the ventral surface, provided with prominent glandular cushions, is facing out (Fig. 19A–C); the posterior extremity is pointed. The creeping sole is as wide as 72–75% of body width in the pre-pharyngeal region (n = 3). The mouth is positioned at a distance from the anterior extremity equal to 63–67% of body length, and the gonopore is 72–78% (n = 3).

The dorsal coloration in live specimens consists of a light ivory (RAL 1015) background color, with numerous sepia brown (RAL 8014) spots which are more (F4218) or less (F4217) merged with each other, with the latter situation presenting a somewhat homogeneous aspect. A midline with the background color may extend along the body length (paratype F4217) or is restricted to the anterior region of the body (paratype F4261). The spots extend to the body sides, where they are scattered so as to create an irregular bordering line, followed by the background color of the sides of the body. A curled anterior extremity is red orange (RAL 2001). The ventral surface is pale red orange in the cephalic region, and light grey (RAL 7035) in the rest of the body (Fig. 19B).

The eyes are formed by a one-pigmented cup of 46–50 μm in diameter. There are no halos around them. Eyes are absent in the very anterior extremity of the body equivalent to 1% of the body length (F4218). Eyes behind the anterior tip are distributed marginally in a row of two or three eyes and extend along the entire body until the posterior extremity.

Sensory pits are 15 μm deep, distributed ventro-laterally in a uniserial row initiating 0.3 mm behind the anterior extremity (the equivalent of 1% of the body length in paratype F4217), and from the very anterior tip in holotype.

In the pre-pharyngeal region, very abundant rhabditogen gland cells pierce the dorsal and marginal epidermis. These types of cells are scarce on the ventral epidermis; instead, there are gland cells producing erythrophil granules and scarce gland cells secreting cyanophil granules. There is no glandular margin (Fig. 19D).

The cutaneous musculature of the pre-pharyngeal region consists of a subepithelial circular muscle, followed by a diagonal layer with decussate fibers, and a longitudinal muscle organized in bundles (Fig. 19D). This longitudinal muscle is 80–100 μm-thick dorsally and organized in tight bundles with approximately 60–110 fibers each; it is ventrally divided into a 28–30 μm-thick muscle organized in bundles with 10–27 fibers each, and a 55–65 μm-thick muscle sunken into the parenchyma and constituted of bundles with 18–40 fibers each (Fig. 19D). The thickness of the cutaneous muscle coat is 22–25% (n = 3) of the body height. There are three parenchymal muscles in the pre-pharyngeal region, namely a dorsal decussate muscle (46–50 μm thick), transverse supra-intestinal muscle (25–30 μm), and transverse subintestinal muscle (70–75 μm) (n = 3) (Fig. 19D).

The cutaneous and parenchymal musculature is organized in the cephalic region as in *Ch. iheringi*. A portion of the retractor muscle of the head is delta-shaped in a cross-section and ranges between 2–5 mm (or 5–14% of body length) from behind, 1–1.3 mm (2–3%) of the anterior extremity of the body (Fig. 19E), and its thickness equals 36% of the height of the cephalic region. The Muskelgeflecht is 200–210 μm thick (30% of body height). The subneural parenchymal muscle consists of transverse fibers. Glandular cushions are composed of very numerous rhabditogen cells and scarce gland cells produce erythrophil granules (Fig. 19E).

The mouth is located in the middle of the pharyngeal pouch (n = 3) (Fig. 20A). The pharynx is bell-shaped, and has its dorsal insertion shifted posteriorly with the equivalent to 44% of the pharynx length. The esophagus length is 20% of the pharyngeal length. The pharyngeal pouch is lined with a non-ciliated, low epithelium underlain by a one-fiber-thick layer of longitudinal muscle followed by 20 µm-thick layer of circular muscle. The outer pharyngeal epithelium is flat, ciliated, and underlain by a one-fiber-thick longitudinal muscle, followed by a 15 µm-thick muscle with some longitudinal fibers interspersed. The inner pharyngeal epithelium is flat, ciliated, and underlain by a mixed layer of circular muscle with longitudinal muscle (75 μm thick). The pharynx presents numerous erythrophil and xanthophil gland cells interspersed.

The testes are mature and dorsally located under the supra-intestinal transverse muscle layer, mostly placed between the intestinal diverticula. They extend from 12.7 mm (30% of body length, holotype) of the anterior extremity of the body to 0.5 mm before the root of the pharynx. Sperm ducts run immediately above the subintestinal parechymatic muscle layer. The sperm ducts bend dorsally close to the copulatory apparatus, and subsequently penetrate the ventral proximal region of the common muscle coat to open into the respective dilated branch of the prostatic vesicle (Fig. 20B). The prostatic vesicle is divided into two differentiated halves; the anterior half proximally presents a dilated and paired portion oriented vertically which opens into a broadened, dish-shaped section located above the paired portion (Figs 20B, 21A, B). This proximal half is extrabulbar and lined by a columnar-to-cuboidal epithelium which is pierced by gland cells producing xanthophil granules. These gland cells are much more abundant in the dish-shaped portion, and present a reddish appearance; the border of this dish-shaped section of the prostatic vesicle is also pierced by gland cells producing cyanophil granules. The distal half is intrabulbar, and is a straight tube running postero-dorsally to open into the proximal region of the male atrium. This half is lined by a columnar epithelium with a sinuous surface which is pierced along its whole length by gland cells producing cyanophil granules; additionally, gland cells producing xanthophil granules pierce its distal portion. The lining epithelium of the proximal half of the prostatic vesicle is coated by a 20 μm-thick circular muscle; the distal half is coated by a 1 µm-thick circular muscle, followed by a 15 µm-thick longitudinal muscle. The male atrium is the same size as the female atrium, and is divided into a proximal, narrow half and a distal, dilated half with some small folds (Figs 21, 22).

The male atrium is lined by a cuboidal, non-ciliated epithelium, and underlain by a 45–80 µm-thick layer of circular muscle with numerous interspersed longitudinal fibers (n = 3). The proximal half of the atrium receives two types of gland cells, one producing erythrophil granules, and a second type of scarce gland cells producing xanthophil granules; the distal half of the male atrium receives abundant gland cells producing xanthophil granules and the sub-apical portion of the cells of the lining epithelium contains xanthophil granules. The extrabulbar portion of the prostatic vesicle is coated by additional muscle fibers attaching it to the common muscle coat (Figs 21, 22).

The ovaries are mature, ovoid, 190 μm in length, and placed above the ventral nerve plate, and at a distance from the anterior body tip equal to 27% of body length (11.5 mm from anterior tip) (holotype). Ovovitelline ducts emerge from the dorso-lateral aspect of the ovaries and run above the ventral nerve plate. Lateral to the female atrium, the ovovitelline ducts bend medially and dorsally, then unite above the postero-dorsal section of the female atrium (Figs 21, 22). The common glandular ovovitelline duct is outside the common muscle coat, and runs posteriorly, progressively inclining to the ventral side to communicate with the posterior section of the female atrium.

The female atrium is divided into a dilated canal running ventro-anteriorly and outside the common muscle coat, and a distal, funnel-shaped half is widely communicated with the male atrium (Figs 21, 22). The female atrium is lined with a 35 µm high epithelium. This epithelium is pierced by gland cells producing fine erythrophil granules. The subapical portion of lining cells of the distal half of the atrium contains xanthophil granules. The lining epithelium of the female atrium is underlain by a layer of mixed circular and longitudinal fibers 10 µm high.

The common muscle coat is a very dense layer composed by densely packed muscle fibers variously oriented (Figs 21, 22). The length:height ratio of the copulatory apparatus enveloped by the common muscle coat ranges between 2.2–2.7:1 (n = 3).

###### Remarks.

*Choeradoplana riutortae* Lago-Barcia & Carbayo, sp. nov. matches all diagnostic characteristics of *Choeradoplana*. The external dorsal coloration resembles 11 species inside the genus with the background color being brownish with dark black or dark brown spots over it, namely *Choeradoplana abaiba*, *Ch. agua*, *Ch. banga*, *Ch. benyai*, *Ch. bocaina*, *Ch. cyanoatria*, *Ch. longivesicula*, *Ch. pucupucu*, and the herein described *Ch. onae* Lago-Barcia & Carbayo, sp. nov., *Ch. eudoxiae* Silva & Carbayo, sp. nov. and *Ch. claudioi* Lago-Barcia & Carbayo, sp. nov. However, none of them present the prominent cushions found in this species, nor the conspicuous red-orange coloration of the cephalic region.

With respect to the internal morphology, *Ch. riutortae* Lago-Barcia & Carbayo, sp. nov. is only similar to *Ch. onae* Lago-Barcia & Carbayo, sp. nov., *Ch. bocaina* and *Ch. claudioi* Lago-Barcia & Carbayo, sp. nov. in that the extrabulbar section of the prostatic vesicle is dish-shaped. However, the female atrium in *Ch. claudioi* Lago-Barcia & Carbayo, sp. nov. is partially below the male one (vs. behind, in *Ch. riutortae* Lago-Barcia & Carbayo, sp. nov.), whereas the male atrium in *Ch. onae* Lago-Barcia & Carbayo, sp. nov. has the same height along its length, (vs. a proximal, narrow half, and a distal, widened half). Finally, the male atrium in *Ch. bocaina* is 3× as long as the female, whereas this ratio is 1.2 in *Ch. riutortae* Lago-Barcia & Carbayo, sp. nov.

## Discussion

The monophyletic origin of the genus *Choeradoplana* was first demonstrated by Carbayo et al. (2013), and subsequently by Lemos et al. (2014) and Carbayo et al. (2018) with additional terminals and species. All species were recovered as monophyletic, perhaps with the exception of *Ch. bocaina*. The clade which includes the three type specimens of *Ch. bocaina* also houses two individuals not studied morphologically which were collected in the Reserva Biológica Augusto Ruschi, ES (500 km distance from the type locality). These two individuals are terminals with long internal branches. In agreement with this view, Carbayo et al. (2018) found uneven molecular delimitations of species depending on the method applied. Interestingly, although the species interrelationships are poorly supported, the species composition of the two large clades is similar to the phylogeny inferred from five mitochondrial and nuclear concatenated genes by Carbayo et al. (2018).

*Choeradoplana* is one among the few geoplanid genera which can be recognized through the body shape. The cephalic region in this genus is typically rolled up and the ventral surface of it is provided with glandular cushions, separated by a longitudinal groove. However, as shown above, the cephalic region of *Ch. albonigra* and *Ch. eudoxiae* Silva & Carbayo, sp. nov. lack the glandular cushions and it is not expanded laterally. In this respect, these two species cannot be distinguished from *Cephaloflexa*. Moreover, the anterior third of the body in *Ch. albonigra* narrows very gradually as in *Cephaloflexa*. Therefore, assignment to a genus must be based on internal aspects of these two species.

Another interesting observation is the distribution of the sensory pits. Following the diagnosis of *Choeradoplana*, these pits occur along a variable portion of the anterior region of the body, but they are absent in the apex (a feature also shared with *Cephaloflexa*). However, sensory pits in *Ch. riutortae* Lago-Barcia & Carbayo, sp. nov. contour the anterior extremity in one of the two examined specimens. Since the sensory pits in the apex are not present in all specimens, it can be interpreted as a deviant individual situation.

The diagnosis of *Choeradoplana* was emended by Froehlich (1955), Ogren and Kawakatsu (1990), and Carbayo and Froehlich (2012) and as a consequence of the morphological variations reported in this paper, we propose to emend the genus as follows (emendation underlined):

Geoplaninae of elongated subcylindrical body. The cephalic region is kept rolled up and backwards; this region usually presents two glandular cushions, ventrally separated by a longitudinal groove. The cephalic region may be laterally dilated giving rise to a “neck” which differentiates this region from the remaining body. Eyes absent in the apex. Sensory pits absent in the apex. Broad creeping sole, more than one-third of body width. Strong cutaneous longitudinal muscles partially sunken into the parenchyma, exclusively ventrally or, more rarely, ventrally and dorsally too. Anteriorly all sunken ventral longitudinal fibers are medially concentrated, constituting the retractor unroller of the cephalic extremity. Bodies of rhabditogen cells located between the retractor and the epidermis. Common glandular ovovitelline duct approaching female genital canal dorsally from anterior direction, more rarely approaching behind the female atrium from the ventral direction.

Four of the six species studied in this paper (not *Ch. albonigra* and *Ch. eudoxiae* Silva & Carbayo, sp. nov.) present the external and internal characteristics of the genus *Choeradoplana*. The morphological diagnostic characteristics of this genus are remarkably heterogeneous in attributes found to be stable within other geoplanin genera. In addition to the variable shape of the cephalic region (discussed above), the longitudinal dorsal cutaneous musculature in *Choeradoplana* can be sunken (*Ch. gladismariae* Carbayo & Froehlich, 2012), a penis papilla can be present (*Ch. crassiphalla*, *Ch. benyai*, *Ch. marthae*), an inverted penis may also occur (*Ch. minima* Lemos & Leal-Zanchet, 2014), the prostatic vesicle may be intrabulbar (several species), the female genital canal can approach the female atrium behind it from the ventral direction (*Ch. banga*). Thus, we have increased the number of morphological variations seen in the genus in the present study, with the most remarkable being the ventral glandular cushions may be reduced or even absent, as in *Ch. albonigra* and *Ch. eudoxiae* Silva & Carbayo, sp. nov. and the anterior third of the body may become progressively thinner towards the anterior tip, as in *Ch. albonigra*. Therefore, one cannot trust the shape of the cephalic region for assigning a species with the head rolled-up to a genus. Instead, histological sections should be examined.

Despite the great variability in the diagnostic features, the genus can still be diagnosed by the following two exclusive attributes among Geoplanids: muscle retractor of the cephalic region delta-shaped in the cross-section and bodies of rhabditogen cells piercing the cephalic ventral epidermis are located between the retractor and the epidermis.

## Supplementary Material

XML Treatment for Choeradoplana tristriata

XML Treatment for Choeradoplana albonigra

XML Treatment for Choeradoplana eudoxiae

XML Treatment for Choeradoplana claudioi

XML Treatment for Choeradoplana onae

XML Treatment for Choeradoplana riutortae
